# Separating the details while maintaining the story: within event episodic integration and across event semantic contiguity in memory

**DOI:** 10.3389/fpsyg.2026.1758774

**Published:** 2026-04-28

**Authors:** Rujuta Pradhan, Mohsen Davoudkhani, Alexia Bouslog, Trase McQueen, Heather Bailey

**Affiliations:** Department of Psychological Sciences, Kansas State University, Manhattan, KS, United States

**Keywords:** aging and memory, episodic memory, semantic memory, within and across events, event memory

## Abstract

The *within* > *across* effect refers to improved memory for information cued from within the same event compared to across events. Although this effect has been demonstrated across various measures of event memory, including temporal order and content-based recall, prior work has primarily relied on binary measures of memory accuracy (i.e., correct/incorrect) and has not evaluated what type of information people recall, such as episodic and semantic details. Further, given that older adults often experience deficits in episodic memory but preserve semantic memory, it remains unclear whether the within > across effect reflects the same underlying processes across memory types and age groups. In two experiments, younger (18–33 years) and older adults (65–85 years) viewed a partial episode of *BBC’s Sherlock* and later completed a cued-recall task. Cue clips were drawn from either the same event (within-event) or an event preceding the target responses (across-event). Participants were asked to recall as many details as possible about the scene following the cue. Experiment 1 presented cues in the narrative order, whereas Experiment 2 randomized cue order to control for schema driven recall. Responses were scored for episodic and semantic detail using a modified version of Levine’s autobiographical memory scoring protocol (Levine et al., 2002). The within > across effect was consistently not observed for episodic details except for older adults in Experiment 1. Instead, a reversed effect (within < across) was seen for gist details across both experiments and age groups suggesting a potential mechanism which helps to maintain contiguity in narrative for otherwise temporally separated event information.

## 1 Introduction

Even though our experience of the environment is continuous, automatic perceptual processes break this experience into a series of discrete events, each segmented from the next by event boundaries (Zacks et al., 2001; Zacks et al., 2007; Zacks, 2020). This process of event segmentation not only shapes how we interpret ongoing experience but also structures memory for these events (Kurby and Zacks, 2008; Swallow et al., 2009; Ezzyat and Davachi, 2011; Pettijohn et al., 2016; Pettijohn and Radvansky, 2016, 2018; Smith et al., 2023; Pu et al., 2022; Xiang et al., 2025). A key outcome of this structuring is the well-documented *within* > *across effect* in event memory: people are more likely to recall information that co-occurs within a single event than information separated into two events by an event boundary (DuBrow and Davachi, 2013, 2014, 2016; Ezzyat and Davachi, 2011; Pettijohn and Radvansky, 2018; Heusser et al., 2018; Clewett et al., 2019; Davis et al., 2021; Davis and Campbell, 2023; Pu et al., 2022; Henderson and Campbell, 2023, but see Wen and Egner, 2022 and Riegel et al., 2025; Henderson et al., 2025).

**FIGURE 1:**
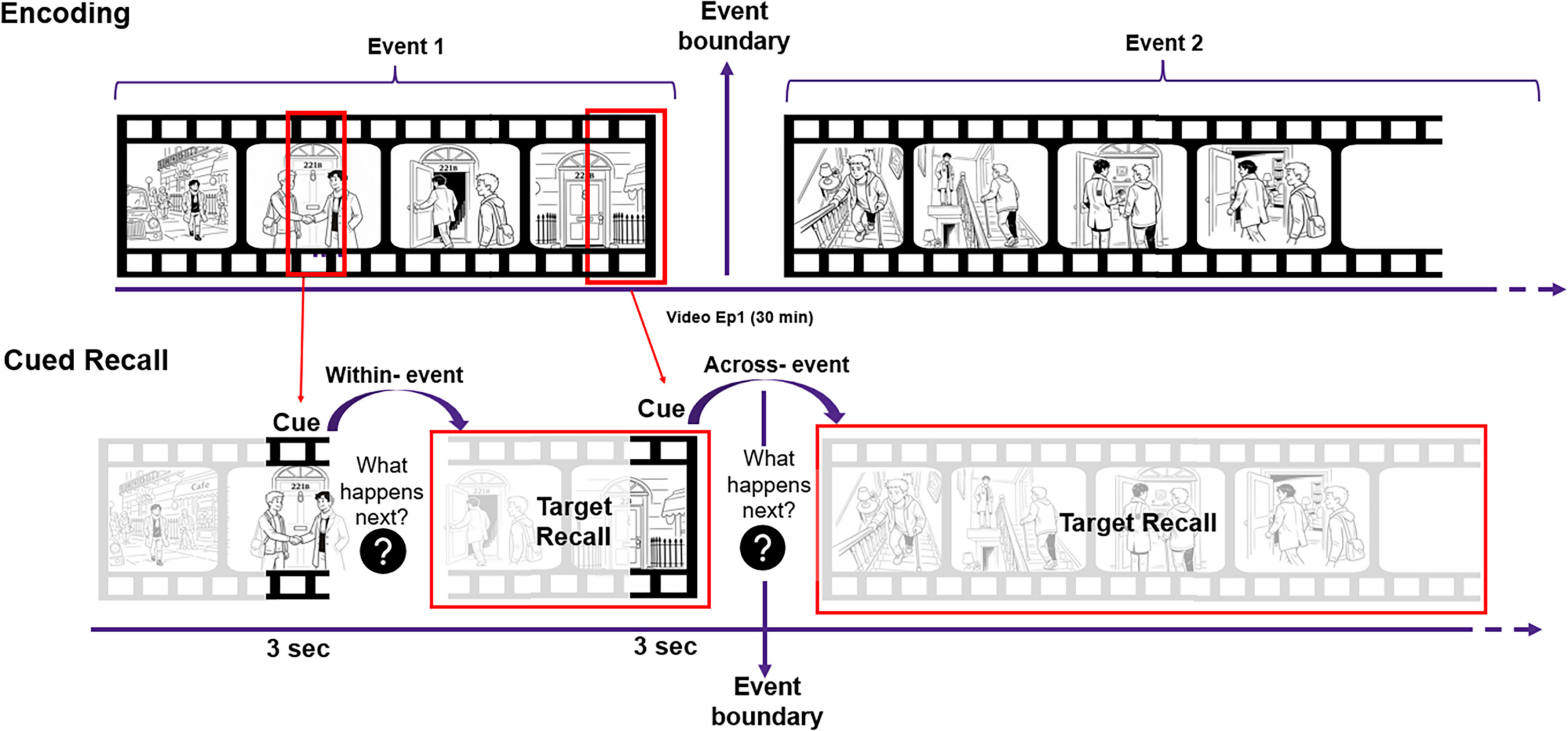
Schematic of the experimental procedure. The ‘Encoding’ panel depicts the separation of the continuous visual stream of information into two distinct events by the occurrence of an event boundary. Both types (within and across) cue clips depicted are extracted from the same event. The ‘Cued Recall’ panel indicates that the identity of the cue clips as a within cue clip and across cue clip is established by the expected location of the to-be recalled target information. Source images of the Figure 1 were generated with ChatGPT (OpenAI, 2026), GPT-5.4 Thinking using OpenAI’s image-generation tools and integrated into the final artwork by author (see Supplementary Data 2 for additional details on prompts used to generate images).

Figure 1 depicts how the within > across effect manifests in cued recall task for dynamic naturalistic events. During encoding of a continuous video, the perception of an event boundary segments the experience into two distinct events. During a later memory test, when participants are presented with a brief (3 s) video cue and asked what occurs next in the video, their accuracy for recalling the correct occurrence is higher when the cue and the to be recalled occurrence (target) belong to the same event (within-event condition) as compared to when the cue and target are separated by an event boundary into different events (across-event condition).

This effect has been demonstrated across a variety of stimuli, including text passages (Ezzyat and Davachi, 2011; Davis and Campbell, 2023; Smith et al., 2023), image or item sequences (DuBrow and Davachi, 2013, 2014, 2016; Ezzyat and Davachi, 2014; Heusser et al., 2018; Xiang et al., 2025), and movies (Henderson and Campbell, 2023; Davis et al., 2021). Further, it influences memory both for when events occur (temporal order information; DuBrow and Davachi, 2013, 2014, 2016; Clewett and Davachi, 2017; Heusser et al., 2018; Xiang et al., 2025) and memory for what happens within an event (content information; Ezzyat and Davachi, 2011; Davis et al., 2021; Davis and Campbell, 2023; Smith et al., 2023; Henderson and Campbell, 2023) The consistency of this effect across representational levels supports the view that information within an event is integrated, whereas information spanning an event boundary is separated (DuBrow and Davachi, 2013, 2016).

Importantly, despite well documented age-related changes in how people perceive, structure and remember events, studies examining event structure observe no age-related differences in the magnitude of the within > across effect (Davis et al., 2021; Davis and Campbell, 2023). However, the existing studies on this effect focus exclusively on *global* memory in cued recall tasks—-whether the cue leads to remembering or forgetting the event—-without distinguishing the type of information being retrieved by young and older adults. This is particularly important to consider given that real-world events are complex and multidimensional, comprised of information that may be differentially processed across age groups. Specifically, event memories include both episodic and gist-based details: episodic representations capture perceptual information such as people, places, and objects, while gist-based representations capture more conceptual information, such as inferred goals or contexts. Thus, the preserved within > across effect in older adults found in prior studies may obscure age-related differences in the type of information supporting event memory.

Given that older adults show selective impairments in remembering episodic details but preserved memory for gist details (Diamond and Levine, 2020, Davis et al., 2021; Greene and Naveh-Benjamin, 2023; Fenerci et al., 2024), they may rely on compensatory gist-based strategies. If this were true, then the overall magnitude of cued recall accuracy may not differ between age groups despite differences in the underlying memory content. Thus, age-related differences afford a valuable opportunity to understand how multiple contextual dimensions (perceptual, semantic, temporal) work together to structure event representations. Because these dimensions vary in how effectively they support information binding in young vs. older adults, age-related patterns can reveal which contextual dimensions are most central for integrating event information across the lifespan.

Taken together, these considerations highlight the need to move beyond global measures of recall and examine *what* kinds of information are driving the within > across effect in each age group. Therefore, the current study examined (1) whether the within > across effect is observed for both episodic and gist-based details in young adults, and (2) whether the direction of this effect for detail types is consistent across age groups. We conducted two experiments, in which participants encoded a 35-min video of the first episode of BBC’s Sherlock. Their memory for pre-defined segments of the video was tested in a cued recall task, in which each cue and the subsequent to-be-recalled segment of the video (i.e., target) either belonged to the same event (within-event cue) or the cue and target belonged to adjacent events, separated by an event boundary (across-event cue). In Experiment 1, the cue clips on the cued recall task were presented in the same order as the video, whereas in Experiment 2, the order of presentation of the cue clips was randomized to replicate the results. First, to assess the within > across effect, we determined which target events were remembered or forgotten. Second, we analyzed only the remembered events to determine how many episodic and gist-based details were recalled in the within-event and across-event conditions, using an adapted version of Levine’s autobiographical memory scoring rubric (Levine et al., 2002) to score recent verifiable memories.

Considering both episodic and semantic information is encoded within a shared event structure, one potential outcome is the observation of a within > across effect for both episodic and gist-based details in both age groups. However, episodic information—such as people, objects, and locations—is likely more tightly bound to specific moments in time and space and thus may be more sensitive to event boundaries, producing a stronger within > across effect. In contrast, semantic or gist-based details—such as concepts, inferred goals, or broader contextual information—are more abstract and may be integrated across multiple events, potentially reducing sensitivity to event boundaries.

Consistent with this proposition, Ding and Zacks (2025) demonstrated that semantic structure can shape memory across event boundaries in verbal narrative contexts. In their study, actions and descriptions were embedded within coarse and fine-grained event structures that either followed or violated a restricted schematic order at each level. During a recency discrimination task for fine-grained actions (i.e., determining which action occurred most recently), participants exhibited a reversed within > across effect; better across event order accuracy than within; when coarse-level events followed a restricted schematic (semantic) order. These findings suggest that individuals can maintain detailed episodic information about specific events while simultaneously encoding semantic information that spans across events. Based on these findings, a second potential outcome is that semantic or gist-based information may show a reduced—or even a reversed—within > across effect, even when episodic details continue to exhibit sensitivity to event boundaries (within > across effect).

Finally, because older adults show deficits in episodic memory, they may rely more on gist-based features of a narrative to guide their recall. Thus, they may show preserved within > across effect or an effect direction consistent with younger adults for semantic information but reduced effect for episodic details compared to younger adults.

## 2 Experiment 1

### 2.1 Methods

#### 2.1.1 Participants

Thirty-nine young (Mean age: 24.59, range: 19–33 years; Mean education: 16.72, Females: 22) and 42 older adults (Mean age: 76.19, range: 67–85 years; Mean education: 16.13, Females: 31) participated in the study. Two older adults did not complete the experiment, and 2 other participants were excluded from analysis who were categorized as having Mild Cognitive Impairment (MCI) based on their Clinical Dementia Rating (CDR) score. Data is reported for 39 young adults (Mean age: 24.6, range: 19–33 years; Mean education: 16.7 years, Females: 22) and 38 healthy older adults (Mean age: 75.8, range: 67–85 years; Mean education: 16.3 years, Females: 29).

Sample size for the experiment was calculated by first transforming the effect size obtained for within-across trials from previous literature (η^2^ = 0.328, Experiment 2, Henderson and Campbell, 2023) to reflect an effect size appropriate for regression models (*f*^2^ = 0.488) using the function “*eta2_to_f2*” function in the *effectsize* package version 1.0.1 (Ben-Shachar et al., 2020) in RStudio (Posit Team, 2025) using R version 4.1.2 (R Core Team, 2021). The pwr.p.test in the “pwr” package version 1.3.0 (Champely, 2020) in RStudio estimated a sample size of 34 participants in each group for the effect size (*f*^2^) = 0.488, α = 0.05, and power = 0.8.

The young adults were recruited from the University of Kansas Medical Center (KUMC) and Kansas State University campuses. The older adults were recruited through the University of Kansas Alzheimer’s Disease Clinic’s Recruitment Core (ADRC) database of participants. Only older adults with a CDR score of 0 (indicating no sign of dementia), and no medical, neurological or psychiatric conditions as evaluated by pre-screening procedures were included in the study. Participants were not excluded based on their prior knowledge of the Sherlock franchise or knowledge/exposure to this specific episode of Sherlock. See Table 1 for the participant demographic information from both experiments.

**TABLE 1:** Demographic characteristics of participants.

	Experiment 1		Experiment 2	
Demographic & tests	Young adult (*N* = 39)	Older adult (*N* = 42)	Young adult (*N* = 42)	Older adult (*N* = 42)
Gender				
Male	17	9	17	14
Female	22	29	25	28
Age				
Mean (SD)	24.59 (4.06)	75.84 (4.60)	25.78 (4.35)	71.64 (4.50)
Min, Max	19, 33	67, 85	18, 35	60, 85
Education (years)				
Mean (SD)	16.72 (2.52)	16.27 (3.72)	18.1 (2.14)	17.7 (2.32)
Min, Max	13, 23	12, 28	14, 22	13, 22
Letter comparison				
Mean (SD)	23.26 (3.31)	15.15 (3.35)	–	–
Pattern comparison				
Mean (SD)	39.23 (7.05)	23.95 (4.45)	48.7 (10.87)	49.9 (10.07)
Vocabulary				
Mean (SD)	0.512 (0.11)	0.596 (0.14)	37.3 (10.14)	50.5 (7.08)

Experiments 1 and 2 use different platforms to assess processing speed and vocabulary knowledge, resulting in measures that were captured on different scales. Experiment 1 Vocabulary reflects the Mean and SD for proportion of items correct, while Experiment 2 reports the Mean and SD for number of items correct.

All participants had normal or corrected to normal vision. Participants with hearing difficulties or hearing prosthesis were excluded from the study due to the audio-visual nature of the stimuli. As this was part of a larger neuroimaging study (fMRI analyses reported in manuscript under preparation), both old and young participants were compensated with a monetary reward of $100, and an additional $50 was provided for travel expenses if they drove more than 100 miles to KUMC. The study was approved by the Institutional review board at KUMC.

#### 2.1.2 Materials

##### 2.1.2.1 Stimuli

The audio-visual stimulus used for encoding was a 35-min video of the first episode of BBC’s Sherlock titled “Study in Pink.” To identify event boundaries in this clip, an independent sample of 50 participants (Mean age: 36.5; Mean education: 15.34; Female: 17) viewed and segmented the clip via the online platform Prolific (www.prolific.com, see Supplementary material 1 for segmentation task details). Since the current paper reports only results from the behavioral cued recall task, scanner details are not included in this paper but reported elsewhere.

The 35-min video clip was edited to remove the portion of the title song and credits (32 s) before the main experiment. The time location of the event boundaries and event middles were adjusted accordingly. The video was projected over a screen inside the fMRI scanner. The experiment was conducted using *PsychoPy* (version 2022.2.5; Peirce et al., 2019).

###### 2.1.2.1.1 Cued recall clips

For the cued recall task, 20 video clips of 3 seconds duration were extracted from the 35-min video. Seven of these clips were extracted such that the duration between their offset and the following event boundary was 5 s. These were defined as across-event cues (*n* = 7) because the clip ended just before an event boundary; thus, the target responses to be recalled were from the next event. Twelve clips had an onset that occurred earlier in an event and, thus, the target recall response was information from the same event as the cue clip. One cue clip was omitted from all further analysis since it was ambiguous and could not correctly be classified as either within/across.

##### 2.1.2.2 Other cognitive tasks

Participants in Experiment 1 also completed processing speed tasks such as letter comparison and pattern comparison as well as the Shipley–Hartford vocabulary test to test for vocabulary knowledge (see Supplementary material 2 for details and Table 1 for participants’ performance).

#### 2.1.3 Procedure

All participants underwent health screenings to ensure MRI safety and general study eligibility requirements were met before participation. First, a short pre-screening survey was completed. If eligible, they were then invited to complete a more in-depth screening over the phone to assess any current medical, neurological, or psychiatric symptoms, as well as non-MR safe medical implants. On the day of the main experiment, participants indicated their written informed consent. They then watched the Sherlock video inside an fMRI scanner while undergoing brain imaging. No behavioral responses were made by participants during the scan. Following the scan, participants completed the free recall task, followed by the cued recall task, and then the letter comparison, pattern comparison and vocabulary knowledge outside the scanner. For the cued recall task, participants typed their response onscreen after viewing each cue clip. This was an untimed task. Finally, participants reported their familiarity with the episode of *Sherlock* (see Supplementary Table 2.1), were debriefed and then received compensation.

##### 2.1.3.1 Cued recall scoring procedure

In both experiments, participants’ responses to the 19 cued recall prompts were scored according to two types of analyses. The first analysis involved the binary scoring of each response as either remembered (1) or forgotten (0). The second analysis focused only on the correctly recalled responses to assess the proportion of episodic vs. semantic details recalled (Levine et al., 2002) by the participants.

For the binary scoring of responses, a rubric of correct responses was developed for each cue (see Supplementary Table 3.1). The correct response described all the actions/occurrences within each event. A score of 1 was assigned to a response if the participant mentioned any of the actions or details included in the correct response rubric. Only the responses that were scored as remembered in the binary analysis were further scored for the proportion of episodic and semantic details recalled. To analyze the number of episodic (Internal) and gist-based (External) details in participant’s response, we adapted the scoring scheme developed by Levine et al. (2002) to distinguish between these two types of details (see Supplementary material 4 for details) in recall of recent verifiable memories.

**FIGURE 2:**
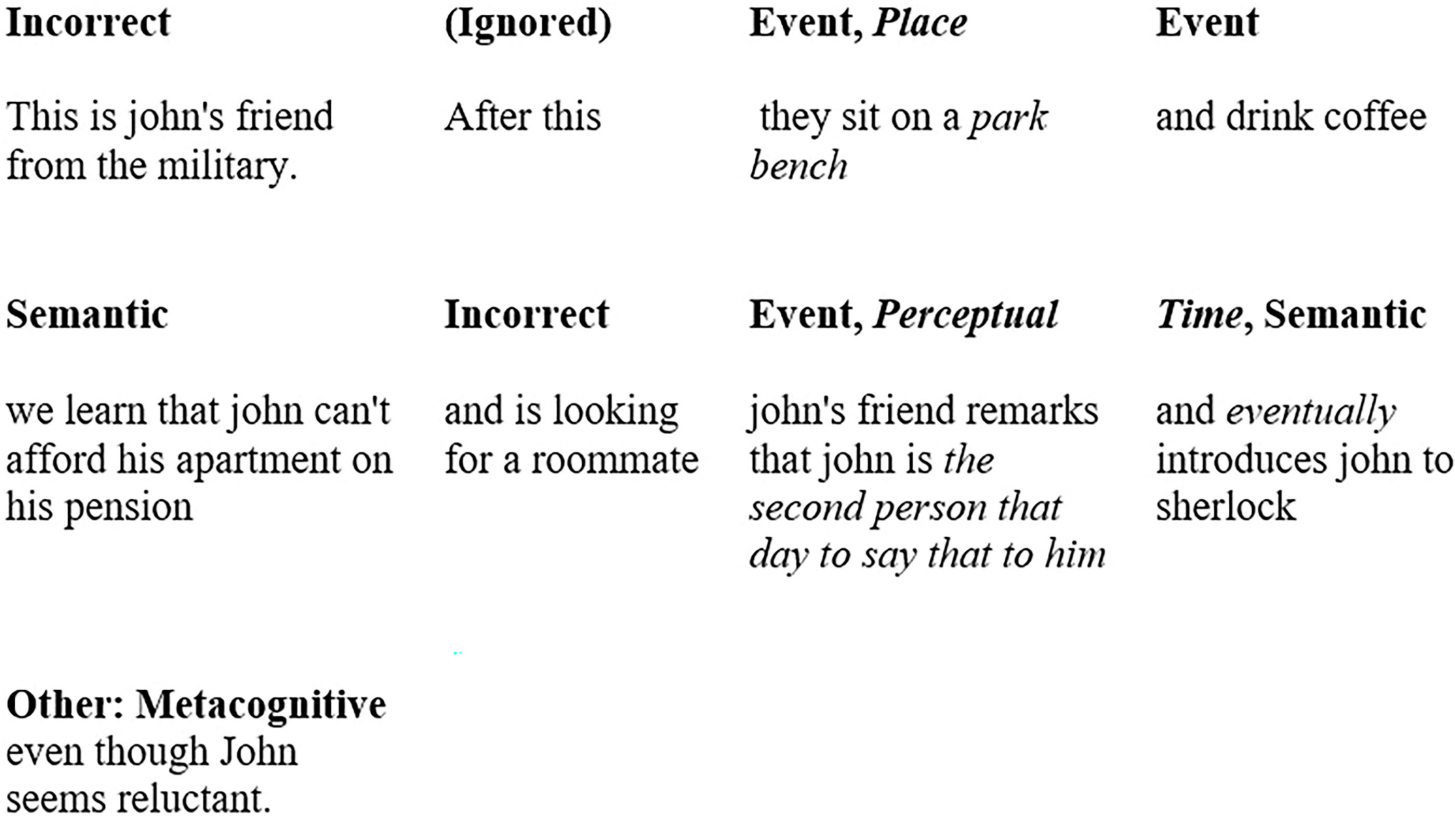
An example of scoring a participant’s response according to episodic and gist-based details. The first clause is categorized as “Incorrect” because the character is John’s friend from college. Incorrect responses do not belong to either episodic or gist-based details. “After this” or “After the clip” is used by participants as a springboard phrase to begin recall and not indicative of time. This phrasing was ignored in scoring. For the same clause being scored in two categories, the category and the words in the clause indicative of that category are in *italics* font.

The final detail categories were “event,” “time,” “place,” “emotion/thought,” “perceptual/sensory detail” included under internal/episodic memory and “semantic details,” “repetition,” “others: Metacognitive/editorial statements,” and “guess responses” included under external/semantic memory. See Table 2 for a full list of categories and their definitions. See Figure 2 for an example of a participant’s response being split into clauses and scored under each category. Additionally, there was an “Incorrect” category added which was not scored under either the episodic or semantic detail, but which included information that did not occur in the target event.

**TABLE 2:** Rubric for categorizing remembered responses into episodic or gist details.

Internal/episodic					External/gist			
Event	Time	Place	Perceptual	Thought/emotion	Semantic/concept	Repetition	Other	Guesses
Occurrences/ happenings/ actions	Year/ season/ month/day of week/ time of day/ duration	Localization of an event including the city, street, building, room, part of room	Auditory, olfactory, tactile, taste, visual and visual details, body position, speed of action	Emotional state, thoughts	General knowledge or facts, ongoing events, extended states of being	Unsolicited repetition of details (repetition from the cue clip)	Meta-cognitive statements, editorializing, implications	Derived from a pilot conductedon a group of 10 participants who predicted post-cue event occurrence without watching the main video

Anonymized data from all participants was classified into the detail categories by training the ChatGPT model 4o (OpenAI, 2025). See Supplementary Figure 4.1 for an example cued recall response scored by ChatGPT. Manual and chatbot scoring followed the same procedure (see Supplementary material 4 for details). Intraclass correlation coefficient (ICC; Koo and Li, 2016) calculated for the proportion of details recalled for 806 randomly selected trials (403 of episodic and gist each) scored by ChatGPT and manual scoring by an independent rater was 0.678 (Model = two-way, Type = consistency, CI = 0.63–0.71).

### 2.2 Results and discussion

First, we report the binary (remembered/forgotten) cued recall results. Then, we report the types of details recalled within the correctly remembered responses. This involves two separate analyses: one for the total number of episodic details recalled and one for the gist-based details recalled.

All statistical analysis reported in this paper were conducted using RStudio (Posit Team, 2025) using R version 4.1.2 (R Core Team, 2021). Model comparisons were conducted using the “*anova*” function in the “*car*” package (v.3.0.12). Linear regressions were performed using packages “*lmerTest*” (3.1.3), and “*glmmTMB*” (1.1.10). All *post hoc* analysis was performed using the “pairs” function in the “*emmeans*” package (1.8.9). All plots were generated using packages “*ggpubr*” (0.6.0) and “*sjPlot*” (2.9.0).

All model comparisons were performed using the method of backward elimination. That is, a full interaction model was first specified and compared to reduced models with the above-mentioned function and package. The models contained the between-subjects factor of “Age group” and within-subjects factor for “Cue type” as fixed effects. For all models reported in the paper, models with a correlated random intercept and slope structure to account for slope variance for the fixed effect of Cue type within participants were run and compared to models with only random intercept for participants specified as random effects. The complex random effect structure was retained only for those models where inclusion of the correlated slope intercepts decreased residual variance and did not show singularity fit or convergence issues. Therefore, all analysis includes the most optimal random effects structure afforded by the data.

#### 2.2.1 Cued recall: remembered vs. forgotten

The first level of analysis focused on scoring participants’ cued recall responses to evaluate global memory accuracy (refer to page 11 and Supplementary Table 3.1 for scoring procedure). Overall participants accurately recalled 53.29% (± 0.25 SD) of target events on the cued recall task. Outlier analysis was performed on the remembered vs. forgotten data. Each participant’s mean accuracy was compared against the outlier criteria (mean ± 3 SD), resulting in the removal of one participant whose mean cued recall accuracy was 0. For the remaining participants, mean accuracy from each age group and trial type is shown in Table 3.

**TABLE 3:** Experiment 1: raw cued recall accuracy performance by cue type and age group.

Age group	Cue type	Mean (SD)
Older adults	Across	0.415 (0.18)
	Within	0.361 (0.20)
	Total	0.382 (0.18)
Young adults	Across	0.663(0.24)
	Within	0.696(0.24)
	Total	0.684(0.22)

The binary (remembered/forgotten) data was analyzed using a generalized linear mixed-effects (GLMM) approach with a binomial link function. Cue Type (within/across) and Age group (young/old) were entered as fixed effects. Random effects included a random intercept for Participants to account for participant-related variability and a random intercept for cue clip to model the variability in the within > across effect across clips. The full interaction model [Accuracy ∼ Cue type * Age + (1 | Cue clip) + (1| Participants)] was compared with reduced models to evaluate the model’s structure and obtain the best fit predictors for the observed data. The model with the lowest AIC was one with the fixed effect for Age group [Accuracy ∼ Age group + (1| Cue_clip) + (1| Participant)]. Since we were interested in effects of both Age group and Cue type, we report the results of the additive model here, but the results of the best fit model are reported in Supplementary Tables 3.4, 3.5.

**FIGURE 3:**
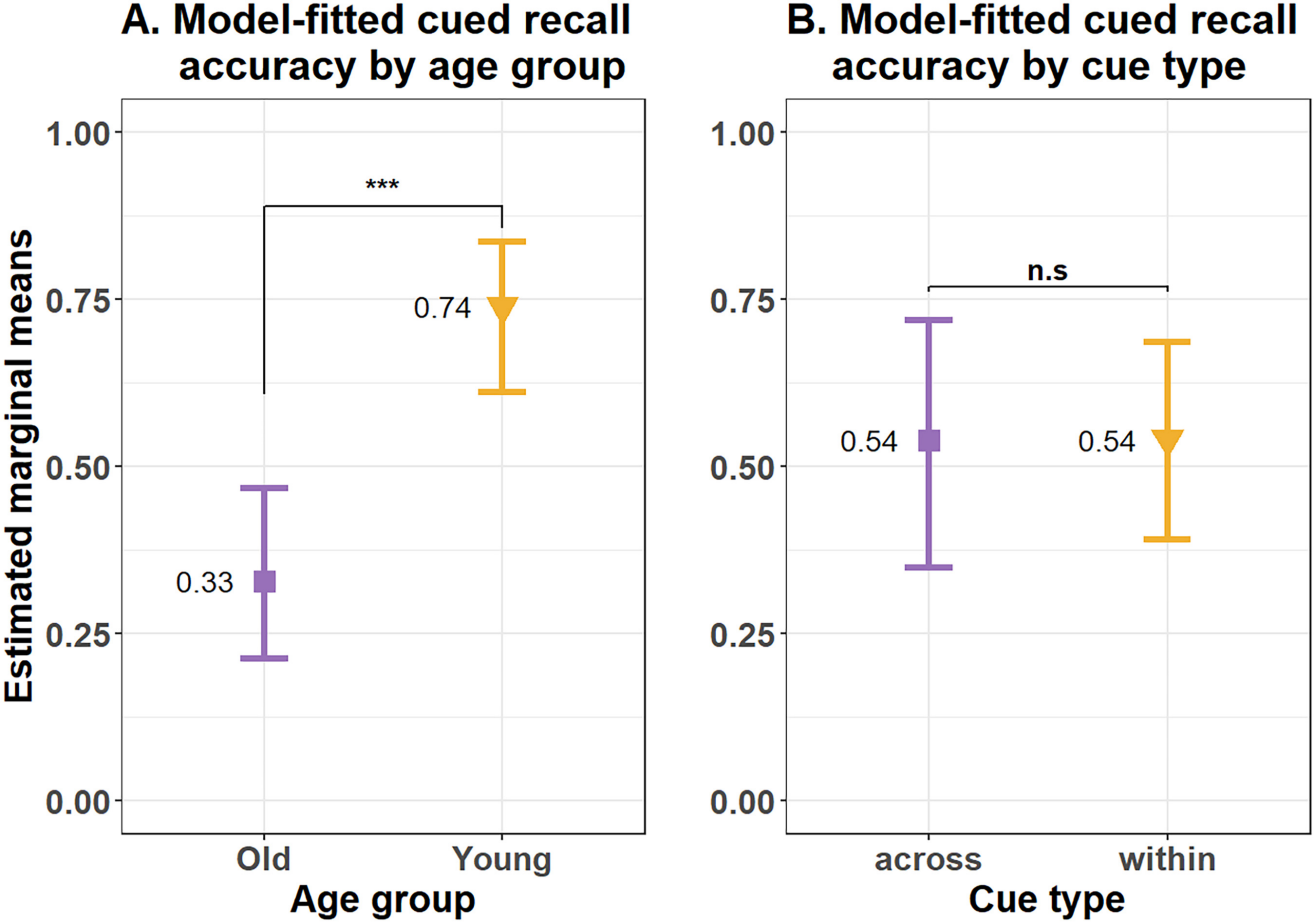
Model predicted mean accuracy by **(A)** age group and **(B)** cue type for the cued recall task (remembered vs. forgotten) in Experiment 1. Error bars represent 95% confidence intervals. ***p < 0.0001; n.s., not significant.

Results of this model showed a significant effect of age group on cued recall accuracy (β = 1.76, SE = 0.27, *z* = 6.47, *p* < 0.001), indicating older adults (estimated marginal mean (emm) = 0.33, SE = 0.07, *df* = Inf, CI = 0.21–0.47) had significantly lower cued recall accuracy as compared to young adults (emm = 0.74, SE = 0.05, *df* = Inf, CI = 0.61–0.84). The estimated marginal means for both the additive model and the best fit model with only Age as a predictor is the same. See Supplementary Tables 3.2, 3.4 for details. But there was no difference in cued recall accuracy as a function of Cue type (β = 0.009, SE = 0.48, *z* value = 0.02, *p* = 0.99), suggesting cues from within the same event (emm = 0.54, SE = 0.07, *df* = Inf, CI = 0.39–0.69) and from across an event boundary (0.54, SE = 0.099, *df* = Inf, CI = 0.35–0.72) were equally helpful in remembering the target event information (see Figure 3).

Therefore, contrary to results of prior studies using naturalistic events along with the cued recall task, we did not replicate the within > across effect in Experiment 1. However, as expected, we observed an age-related deficit in cued recall accuracy.

#### 2.2.2 Cued recall episodic vs. gist

**FIGURE 4:**
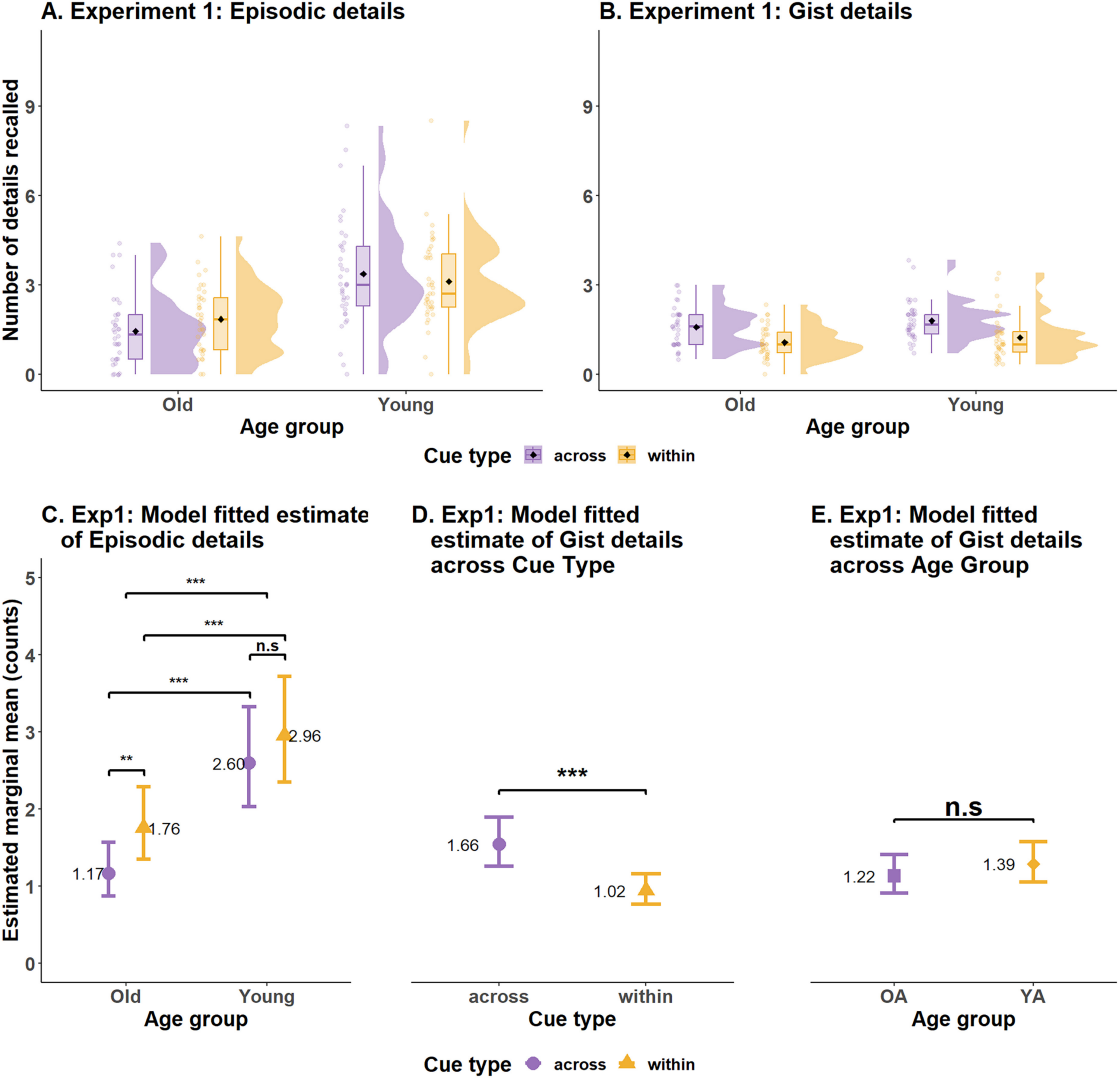
Results of observed and statistically modeled data from Experiment 1. Observed data distribution of average number of episodic details **(A)** and gist details **(B)** recalled by participants. **(C)** Model predicted number of episodic details recalled by both age groups for the two types of cues. **(D)** Model predicted number of gist details recalled for the two cue types averaged over levels of age group. **(E)** Model predicted number of gist details recalled by older and young adults, averaged over levels of cue type. Error bars in **(C–E)** indicate 95% confidence intervals. ***p < 0.0001, **p < 0.001, n.s., not significant.

The second level of analysis for examining the within > across effect focused on different detail types (episodic & gist-based). This scoring was restricted only to the correct responses obtained through binary scoring procedure (53.29% of total trials). Of the correct responses across all participants, the average number of details recalled per trial was 2.12 (±2.23). The raw data (see Table 4) indicated that older adults recalled fewer details per trial (*M* = 1.58, *SD* = ± 1.61) than did young adults (*M* = 2.42, *SD* = ± 2.44). However, young adults also recalled more incorrect details per trial (*M* = 0.26, *SD* = ± 0.24) as compared to older adults (*M* = 0.22, *SD* = ± 0.29). Surprisingly, participants recalled fewer details on the within-event trials (*M* = 2.017, *SD* = ± 2.22) than on the across-event trials (*M* = 2.28, *SD* = ± 2.23; see Figures 4A, B for additional details). Participants also recalled more Incorrect details on the across-event trials (*M* = 0.28, *SD* = ±0.28) than on the within-event trials (*M* = 0.23, *SD* = ±0.24) (see Table 4 for more details).

**TABLE 4:** Experiment 1: mean number of raw episodic vs. gist-based details for within and across cues by age group.

Detail type	Age group	Cue type	Mean count
Episodic	Old	Across	1.634 (2.04)
Episodic	Old	Within	1.976 (2.0)
Episodic	Young	Across	3.507 (2.99)
Episodic	Young	Within	3.353 (2.87)
Gist	Old	Across	1.583 (0.98)
Gist	Old	Within	1.018 (0.92)
Gist	Young	Across	1.805 (1.25)
Gist	Young	Within	1.207 (1.16)
Incorrect	Old	Across	0.22 (0.30)
Incorrect	Old	Within	0.23 (0.28)
Incorrect	Young	Across	0.31 (0.26)
Incorrect	Young	Within	0.23 (0.22)

To understand how age interacts with detail type to modify the within > across effect, two generalized linear mixed analyses were performed: one for the episodic details and another for the gist-based details. These data were analyzed separately because the episodic, but not gist-based, details count data were overdispersed. Thus, a negative binomial distribution was specified in the generalized linear mixed model analysis conducted for the episodic details count data, whereas a “Poisson” distribution was specified while modeling the gist-based details count data. Direct statistical comparison for the total number of details recalled across the two detail types (episodic vs. gist) which was not afforded by the analysis reported below are reported in Supplementary material 6.

##### 2.2.2.1 Episodic details

A full interaction model [Episodic detail counts ∼ Cue type * Age group + (1 + Cue type | Participant) + (1| Cue clip)] with Cue type (within/across) and Age group as fixed effects was specified. A correlated random intercept and slope structure for participants to account for variation due to individual differences and difference in slope of the Cue type and a random intercept for cue clips were specified as random effects in the model. The aforementioned approach for obtaining the best fit model for the observed data showed the interaction model was the one with the lowest AIC and was retained for further analysis.

There was a significant main effect of Age group, β = 0.79, SE = 0.14, *z* = 5.64, *p* < 0.001, with young adults (emm = 2.77, SE = 0.32, CI = 2.21–3.48) recalling more episodic details than older adults (emm = 1.44, SE = 0.18, CI = 1.12–1.85), and a significant main effect of Cue type, β = 0.40, SE = 0.12, *z* = 3.35, *p* < 0.001, with individuals recalling more episodic details on the within-event (emm = 2.28, SE = 0.25, CI = 1.84–2.83) than the across-event trials (emm = 1.75, SE = 0.2, CI = 1.38–2.20). These main effects were qualified by a significant interaction between Cue type and Age group, β = −0.28, SE = 0.13, *z* = −2.07, *p* < 0.05 (see Supplementary Table 4.1 for details).

*Post hoc* analysis was performed by computing estimated marginal means across pairs applying Tukey’s HSD correction for multiple comparisons. Young adults recalled significantly more episodic details than the older adults on both the within-event trials (Young: emm = 2.96, SE = 0.35, df = Inf, CI = 2.35–3.72; Older: emm = 1.76, SE = 0.24, df = Inf, CI = 1.35–2.29), ratio = 0.59, SE = 0.07, df = Inf, *z*.ratio = −4.096, *p* < 0.001 and the across-event trials (Young adults: emm = 2.60, SE = 0.32, df = Inf, CI = 2.032–3.33; Older adults: emm = 1.17, SE = 0.17, df = Inf, CI = 0.88–1.57), ratio = 0.45, SE = 0.06, df = Inf, *z*.ratio = −5.64, *p* < 0.001. Most important, a significant within > across effect for episodic details was only observed in older adults (ratio = 0.665, SE = 0.08, df = Inf, *z*.ratio = −3.35, *p* < 0.01) but not for young adults (ratio = 0.87, SE = 0.07, df = Inf, *z*.ratio = −1.71, *p* = 0.31) (see Supplementary Table 4.2 for emm details). While these results replicate prior literature in that young adults remember more episodic details than older adults (see Figure 4C), they only partially replicate the within > across effect (i.e., we did not observe the effect in young adults).

##### 2.2.2.2 Gist details

The gist-based count data was also modeled using generalized linear mixed models, but the underlying distribution was assumed to be Poisson. Except for this, the modeling procedure for obtaining the best fit model for the data, fixed effects and random effects were all the same as the episodic details models. Model comparison analysis indicated that an additive model [Gist detail counts ∼ Cue type + Age group + (1 + Cue type | Participants) + (1| Cue clip) with the fixed effect for Cue type and Age group, correlated random slope intercept for participants and random intercept for Cue clip had the lowest AIC and was also significantly better (*p* < 0.001) than the full interaction or reduced models.

Although young adults (emm = 1.39, SE = 1.42, df = Inf, CI = 1.13–1.70) remembered greater amount of gist-based details than older adults (emm = 1.22, SE = 0.14, df = Inf, CI = 0.98–1.52), this difference was not statistically significant, β = 0.13, SE = 0.09, *z* = 1.366, *p* = 0.17 (see Supplementary Table 4.3 for details). For the effect of Cue type, interestingly, results showed that participants recalled more gist-based details on the across-event trials (emm = 1.66, SE = 0.17, df = Inf, CI = 1.35–2.03) than on the within-event trials (emm = 1.02, SE = 0.11, df = Inf, CI = 0.83–1.25), β = −0.48, SE = 0.07, *z* = −6.421, *p* < 0.001 (see Figure 4D).

Thus, we observed different age-related patterns across the two detail types, as well as distinct within > across effects for episodic vs. gist-based details. Specifically, we observed an age-related deficit in recalling episodic, but not gist-based, details. This pattern aligns with prior work showing that older adults exhibit declines in episodic memory while largely preserving semantic abilities (Pitts et al., 2022; Smith et al., 2020). Further, for episodic details, we found the expected within > across effect, but only for older adults. In contrast, we observed a strikingly different pattern for gist-based details: participants in both age groups recalled more gist information from across an event than from within an event.

One methodological feature to note here is that the cue clips in Experiment 1 were presented in the same serial order as they occurred in the video during encoding. Prior studies evaluating the within > across effect in narrative stimuli have been mixed in this regard, with some presenting cues sequentially (Davis and Campbell, 2023; Henderson and Campbell, 2023) and others presenting them in random order (Ezzyat and Davachi, 2011). Presenting cues sequentially may have inadvertently activated a schema for the storyline within the episode, thereby facilitating gist-based recall. Such schema activation should, in principle, have uniformly benefited both within- and across gist-based recall; however, we investigated whether this possible confound contributed to the observed pattern of result. We also examined whether these results from Experiment 1 replicate in a different sample. Therefore, in Experiment 2, we retained all experimental procedures and stimuli with the exception that the cue clips were presented in a randomized order during the cued recall task.

## 3 Experiment 2

### 3.1 Methods

#### 3.1.1 Participants

This sample consisted of 42 young adults (Mean age: 26, range: 18–35 years; Mean education: 18.1 years, Females: 25) and 42 older adults (Mean age: 72, range: 60–85 years; Mean education: 17.7 years, Females: 28). All participants were individuals from the local Manhattan community. Young adults were recruited through the Kansas State Cognitive and Neurobiological Approaches to Plasticity (CNAP) Sona system, whereas older adults were recruited using databases maintained by the research laboratory. Participants were required to be cognitively healthy, either native or proficient English speakers, and have normal or corrected-to-normal vision. The study protocol was approved by the Institutional Review Board (IRB No. 11232) of Kansas State University. All participants provided informed consent before participating and were compensated $50 for their participation.

#### 3.1.2 Materials

All stimuli and tasks were identical to those used in Experiment 1, with the following exceptions.

##### 3.1.2.1 Stimuli

The cued recall task included the same cue clips generated for Experiment 1, including the duration as well as the number of within-event and across-event trials. The only difference was in the order of presentation of the cue clips, i.e., cue clips were presented in a random order (rather than in serial order, as in Experiment 1).

##### 3.1.2.2 Other cognitive tasks

Participants in Experiment 2 also completed two additional event memory tests about the *Sherlock* episode (a recognition test and temporal order memory test) as well as a battery of psychometric tests. These data are not reported in this manuscript. But see Supplementary material 5 for task details.

#### 3.1.3 Procedure

After signing a consent form, participants were set up on a 64-channel EEG equipment and taken to a sound-attenuating booth (WhisperRoom, Knoxville, TN), where they received on-screen instructions about the study in a softly illuminated environment. First, participants watched the 1-min 2D animation clip (*Morning!*). Then, participants watched the *Sherlock* video without any specific task requirements while the EEG data was recorded. Afterwards, the EEG cap was removed and then the remaining cognitive assessment tasks were completed. All tasks were administered in the following order, using *PsychoPy* (version 2023.2.3; Peirce et al., 2019) for Free recall, cued recall, recognition memory, and temporal order memory. Next, they completed the NIH Toolbox version 1.7 (2021) assessment. Finally, participants were debriefed about the study’s purpose and thanked for their contribution.

##### 3.1.3.1 Cued recall scoring procedure

The cued recall performance was scored and analyzed using the same procedures described in Experiment 1.

### 3.2 Results and discussion

As with Experiment 1, we first will report the binary (remembered/forgotten) cued recall results. Then, for only the correctly recalled responses, we will report the types of details recalled within the correctly remembered responses, separately for the number of episodic details and the gist-based details. All statistical analysis reported in this paper were conducted using RStudio (Posit Team, 2025, R version 4.5.2) using the same packages as those in Experiment 1 (see page 12 and 13 for details).

#### 3.2.1 Cued recall: remembered vs. forgotten

Mean accuracy for the cued recall task was (*M* = 0.50%, *SD* = ± 0.23, see Table 5 for details). For the first analysis, a generalized linear mixed-effects model (GLMM) with a binomial link function was conducted, including the within-subject factor of Cue type (within-event vs. across-event) and between-subject factor of age group (young vs. older adults) as fixed effects. Random correlated intercept and slope to account for participant variation in intercept and slope across Cue types and a random intercept for clips to account for stimulus-related variability were included as random effects. The full interaction model was compared with reduced models. Although the model with the lowest AIC included only Age Group, we report the additive model [Accuracy ∼ Cue Type + Age Group + (1 | Clip) + (1 + Cue type | Participant)] here to maintain consistency with Experiment 1.

**TABLE 5:** Experiment 2: raw cued recall accuracy performance by cue type and age group.

Age group	Cue type	Mean (SD)
Older adults	Across	0.418 (0.219)
	Within	0.442 (0.241)
	Total	0.434 (0.211)
Young adults	Across	0.529 (0.274)
	Within	0.587 (0.236)
	Total	0.565 (0.226)

**FIGURE 5:**
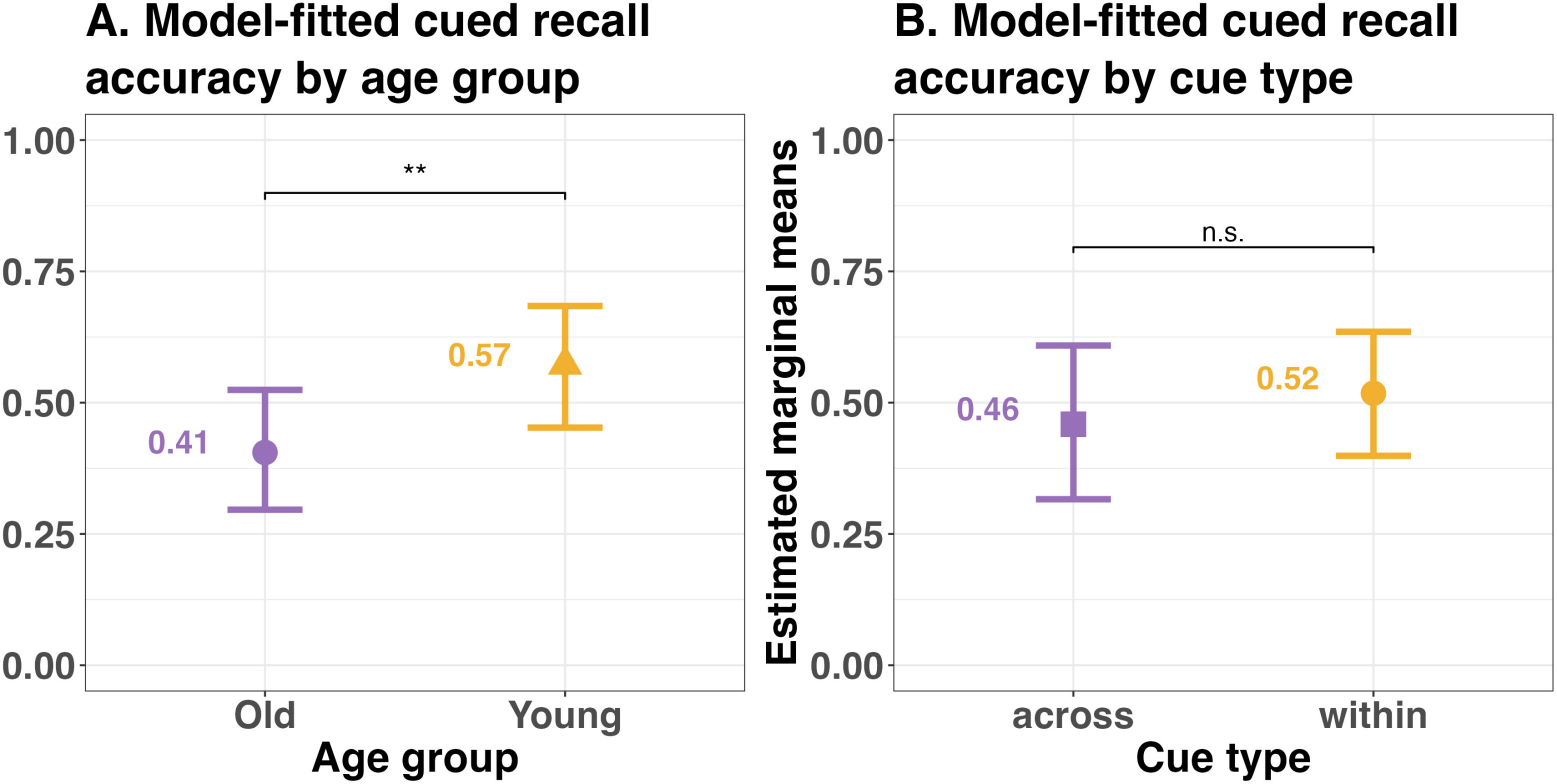
Model predicted mean accuracy by **(A)** age group and **(B)** cue type for the cued recall task (remembered vs. forgotten) in Experiment 2. Error bars represent 95% confidence intervals. **p < 0.01; n.s., not significant.

Results revealed a significant main effect of age group (β = −0.344, SE = 0.123, *z* = −2.79, *p* < 0.01), indicating that older adults showed lower cued recall accuracy than younger adults (see Figure 5A). Model-based estimated marginal means averaged across cue type confirmed this pattern, with lower predicted accuracy for older adults (emm = 0.404, SE = 0.059, CI = 0.295–0.522) relative to younger adults (emm = 0.574, SE = 0.059, CI = 0.455–0.685). However, as in Experiment 1, the main effect of Cue type was not significant (β = −0.117, SE = 0.182, *z* = −0.64, *p* = 0.52). Estimated marginal means, averaged across age group showed comparable recall accuracy for within-event cues (emm = 0.518, SE = 0.061, CI = 0.40–0.635) and across-event cues (emm = 0.459, SE = 0.077, CI = 0.317–0.609; see Figure 5B), indicating no reliable within-event advantage (see Supplementary Tables 5.3, 5.4 for details).

Random effect intercepts accounted for variability across participants (*SD* = 0.87) and clips (*SD* = 0.94). Overall, consistent with Experiment 1, older adults demonstrated lower cued recall accuracy than younger adults, and there was no evidence for a within-event advantage or age-related differences in the magnitude of the cue-type effect.

#### 3.2.2 Cued recall: episodic vs. gist details

**FIGURE 6:**
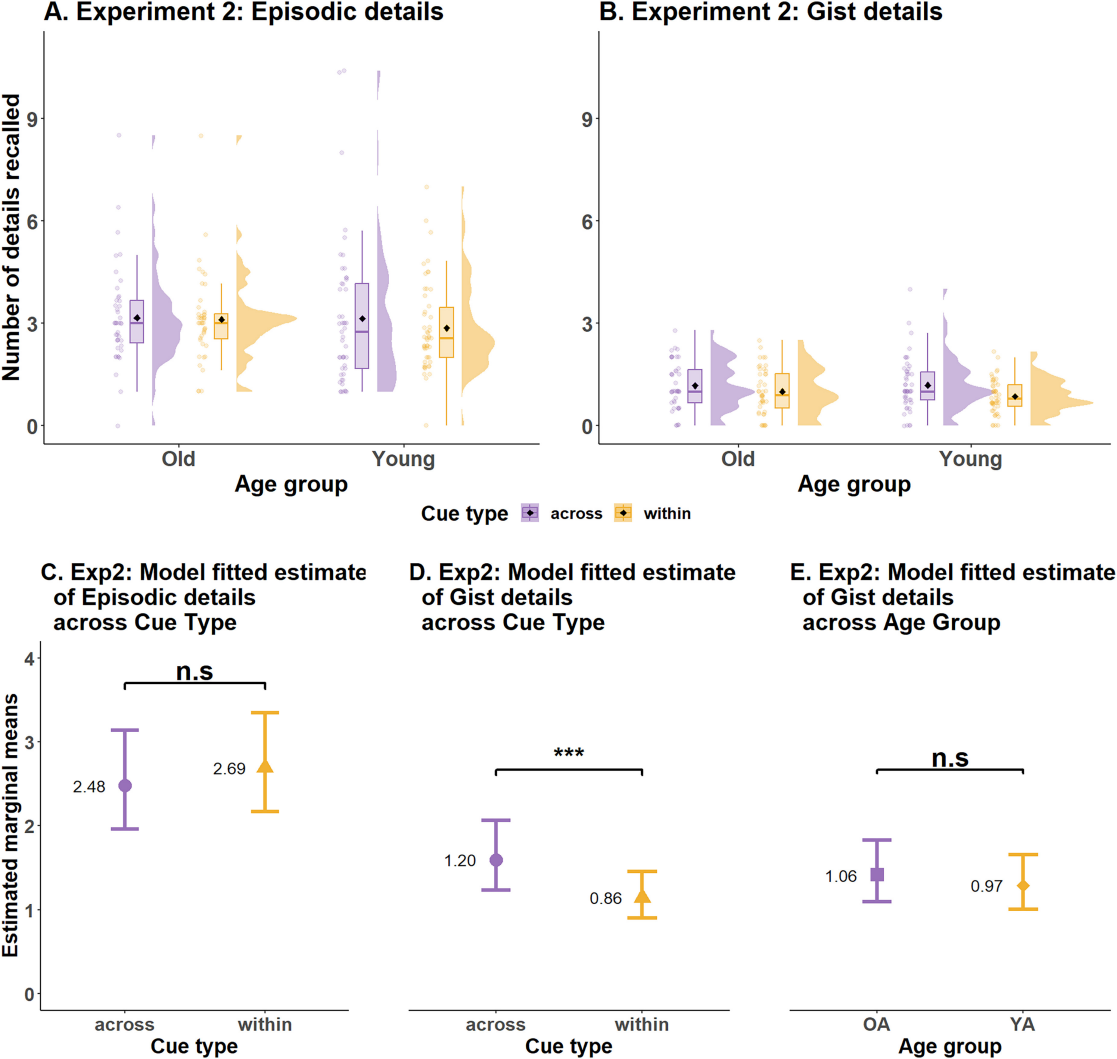
Results of observed and statistically modeled data from Experiment 2. Observed data distribution of average number of episodic details **(A)** and gist details **(B)** recalled by participants. **(C)** Model predicted number of gist details recalled for the two cue types averaged over levels of age group. **(D)** Model predicted number of gist details recalled by older and young adults, averaged over levels of cue type. Error bars in (**C–E**) indicate 95% confidence intervals. ***p < 0.001; n.s., not significant.

Next, we conducted two different generalized linear model analyses: one for episodic details and one for gist-based details only for the remembered trials (*M* = 0.49%). The raw data indicated that the average number of details recalled was (*M* = 2.16, *SD* = ±2.29) details per event (see Table 6 for additional details). On average, older adults reported (*M* = 2.19, *SD* = ±2.17) a similar number of details as did the young adults (*M* = 2.15, *SD* = ±2.36). Across both age groups, the average number of details reported on the across-event trials (*M* = 2.42, *SD* = ±2.70) was greater than the average number of details reported on the within-event trials (*M* = 2.03, *SD* = ± 2.02; see Figures 6A,B for additional details). A similar number of incorrect details were remembered for the across (*M* = 0.05, *SD* = ±0.13) and within-event trials (*M* = 0.08, *SD* = ±0.13) (see Table 6 for details).

**TABLE 6:** Experiment 2: mean number of raw episodic vs. gist-based details for within and across cues by age group.

Detail type	Cue type	Age group	Mean count
Gist	Across	Old	1.244 (1.02)
Gist	Within	Old	1.033 (1.04)
Gist	Across	Young	1.227 (1.01)
Gist	Within	Young	0.887 (0.87)
Episodic	Across	Old	3.429 (2.94)
Episodic	Within	Old	3.191 (2.15)
Episodic	Across	Young	3.746 (3.51)
Episodic	Within	Young	3.060 (2.26)
Incorrect	Across	Old	0.03 (0.07)
Incorrect	Within	Old	0.09 (0.14)
Incorrect	Across	Young	0.06 (0.16)
Incorrect	Within	Young	0.08 (0.13)

##### 3.2.2.1 Episodic details

The full interaction model for the episodic details count data included Cue type, Age group and their interaction as the fixed effects, specifying a negative binomial distribution. A correlated random intercept and slope to account for variability in slope of Cue type by participants and a random intercept for cue clip was included as random effects in the model. Comparisons with reduced models indicated the model with Cue type as fixed effects had the lowest AIC and was significantly different from the full interaction and additive model. Note: the model with fixed effects was compared to null model with only random intercepts specified for participants and cue clip because the model with a more complex random effects structure failed to converge. Results showed no significant difference (β = 0.08, SE = 0.05, *z* = 1.51, *p* = 0.13; see Supplementary Tables 5.6, 5.7 for details) in the number of episodic details recalled in the within (emm = 2.69, SE = 0.30, CI: 2.17–3.34) and across (emm = 2.48, SE = 0.30, CI = 1.96–3.14) cue condition (see Figure 6C). These results are contrary to those observed for episodic details in Experiment 1 where we found a significant within > across effect at least for older adults.

##### 3.2.2.2 Gist details

A generalized linear mixed effects model for the gist-based details count data with the distribution family specified as “Poisson” analysis was performed. The fixed effects and random effects specified for the full interaction model were the same as those for the initial model specified for episodic details. Model comparison showed the model with the fixed effect for Cue type and random intercepts for cue clips and participants [Gist-based counts ∼ Cue type + (1 | Cue clip) + (1 + Cue type| Participant)] had the lowest AIC (2262.5) but it was not significantly different from the additive model including both fixed effects of Age and Cue type (AIC = 2263.6). Since we were interested in the effects of both predictors, the additive model was retained for further analysis.

Similar to Experiment 1, the main effect of Age was not significant, β = −0.09, SE = 0.09, *z* = −0.92, *p* = 0.36, indicating that young adults (emm = 0.969, SE = 0.123, df = Inf, CI = 0.75–1.24) and older adults (emm = 1.062, SE = 0.14, df = Inf, CI = 0.82–1.37) recalled a similar number of gist-based details (see Figure 6E). However, the main effect of Cue type was significant, β = −0.33, SE = 0.09, *z* = −3.68, *p* < 0.001 (see Supplementary Tables 5.8, 5.9 for details). Replicating Experiment 1, participants recalled a greater number of gist-based details on the across-event trials (emm = 1.2, SE = 0.16, df = Inf, CI = 0.92–1.55) as compared to the within-event trials (emm = 0.86, SE = 0.10, df = Inf, CI = 0.67–1.09; see Figure 6D for details).

In summary, results from Experiment 2 closely mirrored those of Experiment 1. We found that participants’ cued recall responses in Experiment 2 did not demonstrate the within > across effect reported in prior studies, in either the global mean accuracy nor in their total number of episodic or gist-based details recalled. Rather, participants showed the reverse effect: they recalled more gist-based details on the across-event trials than on the within-event details.

## 4 General discussion

One of the major goals of the studies reported here was to determine whether the within > across effect—-i.e., the tendency to recall information that co-occurs within a single event more readily than information separated into two events by an event boundary—-is reflected for both episodic and semantic information of a naturalistic event. Specifically, we examined whether event structure strengthens the associative links among both episodic and semantic details that occur within an event, compared to details divided across event boundaries. The second major goal of the current set of studies was to determine whether there are age-related differences in this mnemonic organization of episodic and semantic information, which may explain the overall age-related deficit reported for event and episodic memory (Kurby and Zacks, 2011; Davis and Campbell, 2023).

To achieve this, across two experiments young and older adults watched an episode of BBC’s *Sherlock* and completed a cued recall task. This task consisted of trials in which the cue and target information come from the same event (i.e., within-event trials) or from neighboring events (across-event trials). We reasoned episodic details—-the perceptual components of experience such as time, locations, characters, and objects—-are tightly bound within a temporal context and, by extension, within an event. In contrast, the conceptual or gist-based details—-such as inferences drawn about the characters’ goals and plot—-are more likely to be organized by the schematic context that can span multiple events; as a result, memory for these gist-based details may be less affected by the presence event boundaries. Thus, we expected a stronger within > across effect for episodic details as compared to gist-based details of an event. Further, given that older adults typically experience declines in remembering episodic features and rely more on gist-based features, we expected to find an age-related difference in the magnitude for the within > across for episodic details, with younger adults showing a greater within > across effect than older adults.

First, across both experiments, we observed the expected age-related deficit in overall cued recall performance. We also observed an age-related deficit in the number of episodic details recalled (though this effect was not significant in Experiment 2). However, we found no significant age-related differences in the number of gist-based details recalled. Taken together, these results align with past work which indicates that aging disproportionately affects memory for episodic information, whereas gist-based, semantic information remains relatively preserved (Greene and Naveh-Benjamin, 2023; Davis et al., 2021; Davis and Campbell, 2023; Umanath and Marsh, 2014). This dissociation highlights a shift in the representational content that supports memory performance in older adulthood.

Contrary to our expectations, we did not replicate the well-established within > across effect for the overall accuracy of cued recall task for both Experiments 1 and 2. In fact, we found that neither age group showed this effect. Despite this null effect in the global memory measure, we assessed the within > across effect for both types of details. To do so, only remembered responses were further analyzed to evaluate the number of episodic and gist-based details recalled by participants. Our results show that the occurrence of event boundaries does not uniformly separate all event information into discrete units. A within > across effect was observed in older adult’s memory for episodic details (Experiment 1). However, for gist-based details, this pattern was consistently reversed across both experiments. That is, more gist-details were reported when the cue and target belonged to two different events as compared to when the cue and target belonged to the same event (within < across). This reversal was true for both age groups. The results for episodic details are consistent with the idea that sensory features of an experience—-such as the actions, time, locations and perceptual components of a scene—-are embedded within a shared spatio-temporal context.

One possible explanation for greater gist-based recall across events could be greater causal connectedness between cue and target for across trials. Although we cannot use inferential statistics to test this question without losing power, the descriptive data regarding causal connectedness (Van Den Broek, 1990; Radvansky et al., 2014) between cue and target (see Supplementary Tables 7.1, 7.2 for details) shows an inconsistent pattern of the influence of causality between cue and target and across trial gist-detail recall across the two experiments. Specifically, Experiment 1 shows an inverse relationship while Experiment 2 shows a facilitatory relationship between causal connectedness and across trial gist detail recall. Further studies which manipulate causal connectedness between cue and target to observe its influence on across trial detail recall are needed to better understand this relationship. Since a greater across > within effect for gist details was observed in both Experiments despite the variation in causal connectivity between cue and target, it the effect is not likely dependent on causal connectedness between events.

The reversed pattern observed for gist-based details is also largely inconsistent with prior literature on the within > across effect, with one notable exception. In the study by Wen and Egner (2022) they used the Ezzyat-DuBrow-Davachi (EDD) (Davachi and Murty, 2024) paradigm along with a temporal order task. In this paradigm, participants encode a series of gray-scale images embedded in distinct color frames. The color frame acts as perceptual context for the images. A change in this color context induces an event boundary binding images encoded within the same perceptual context into an event. Wen and Egner (2022) observed a reversed across > within effect when the context (color frame) in which the images were encoded was also provided during the order judgment task. They concluded that the different contexts served as diagnostic cues for serial order, making across-boundary items easier—not harder—to discriminate. This conclusion is consistent with Event Horizon Model’s principle of “superiority of memory for information stored across multiple events in non-competitive attribute retrieval” (Radvansky, 2012).

In the current set of experiments, the memory measure was not serial order but rather recall of gist-based information cued by information embedded in a different context (i.e., event). Under these circumstances, across-event cueing should not facilitate recall unless gist-based information from separate events was associated at some higher level of representation. Thus, the current findings cannot be fully explained by the mechanism proposed by Wen and Egner; instead, they suggest that gist information may be organized across events in a fundamentally different manner than episodic information.

Support for this interpretation comes from recent work demonstrating an across > within effect when participants relied on semantic information spanning event boundaries to perform a recency discrimination task on fine events (Ding and Zacks, 2025). These results raise the possibility that, within hierarchical event structures, semantic information may be associated across events even when event boundaries separate lower-level information. Notably, the pattern observed for gist-based information in the current study aligns more closely with the nested cortical hierarchy observed for neural representation of events (Baldassano et al., 2017). Events are represented at different timescales: sensory regions represent fine-grained short-duration events, whereas higher cortical regions represent coarser, goal-relevant-events—-and event boundaries at these different levels tend to align across the hierarchy. Representations of episodic details in the current study may map onto these fine-grained sensory representations, whereas the gist-based representations correspond to coarse level representations.

Although prior studies have established that perceptual organization of events is preserved in memory (Swallow et al., 2009; Sargent et al., 2013; Pettijohn et al., 2016), the current results further suggest that event memories have at least two levels of representation. The gist-based representation appears to be organized within a schematic framework that helps maintain contiguity and coherence across episodes, even when event boundaries segment episodic information into separate event models. The gist-detail categories include semantic descriptions of event, repetitions from cues or details of the same event, metacognitive or editorial statements reflecting participants’ inferences about the character’s goals, mental states or personality characteristics, and predictive guesses about subsequent events. Except for repetition category, recall in other categories requires participants to construct and maintain an understanding of the larger storyline and goals abstracted from prior events. By contrast, the episodic information may be more tightly bound to temporal context. Further research that experimentally manipulates schema- or gist-level context will be necessary to determine whether these representations are also temporally organized—albeit over longer timescales—or whether they follow a fundamentally different organizational structure.

This opposite pattern of memory advantages for episodic vs. gist details helps explain why the overall cued recall analysis did not show a within > across effect. When episodic and gist-based details were combined, the reversed pattern for gist information canceled out the within-event advantage observed for episodic details. The current findings could indicate that the within > across effect reported in prior studies using global cued-recall accuracy likely reflects the contribution of episodic details alone, masking the distinct organizational principles governing gist-level information. This distinction is especially important for research on age-related changes in event memory, because older adults generally show deficits in episodic memory but relatively preserved semantic memory.

Prior studies have not found age-related differences in the within > across effect when assessing global measures of memory, indicating that the organization of event information is broadly similar in both young and older adults (Davis et al., 2021; Davis and Campbell, 2023). In the current study, however, statistical evidence for the within > across effect for episodic details was observed for only older participants in Experiment 1, although young adults showed the same trend. This pattern runs counter to our prediction that young adults would exhibit a stronger within > across than older adults.

A related null finding was reported by Henderson and Campbell (2023), who did not observe the within > across effect in one of their groups; notably, older adults with lower working memory capacity did not show the effect. They argued that working memory updating supports event segmentation and the binding of within-event information. Because we did not have a measure of memory capacity or updating, we were unable to test this possibility. Future studies evaluating the within > across effect—-particularly in older adults—-should investigate this claim further.

An alternative explanation for the age-related difference observed here is that young and older adults may differ in their perception of event boundaries. Because event boundaries in the video stimuli were identified using a sample composed of primarily young and middle-aged adults, the observed age-related differences may reflect variation in boundary perception rather than differences in memory, *per se*.

Across the two experiments, we did not observe a within > across effect on the cued-recall task for either age group, for the reasons described above. One additional possibility is that the study was underpowered to detect the interactive effects of cue type and age, as sample size calculations were based on the main effect size of cue type. Notably, however, a within > across effect did emerge for episodic-detail memory in older adults, but only in Experiment 1, in which retrieval cues were presented in the same serial order as encoding. The effect was not present when cue order was randomized.

This pattern suggests that older adults’ retention of episodic details was facilitated when the encoding schema was reinstated at retrieval. In this way, the present findings extend prior work showing that older adults rely on prior knowledge to encode novel episodic information (e.g., Umanath and Marsh, 2014), by demonstrating that they also draw on this knowledge structure as a retrieval cue. Finally, the presence of a reversed effect for gist details regardless of whether the event schema was preserved (Experiment 1) or disrupted (Experiment 2), suggests that this pattern does not arise from the binding of semantic details to a schematic context. Instead, it may reflect a distinct way in which semantic information about events is organized.

The within > across effect has been instrumental in advancing our understanding of how information is separated and integrated within event representations. Most studies investigating the organization of event information through this effect have focused on the temporal representation of event information. Therefore, most empirical findings are based on paradigms that use temporal order or temporal judgment tasks (Ezzyat and Davachi, 2011; Diamond and Levine, 2020; Davis et al., 2021; Davis and Campbell, 2023; Henderson and Campbell, 2023; Ding and Zacks, 2025). Results from these tasks are well accounted for by theoretical frameworks such as the Temporal Context Model (TCM; Howard and Kahana, 2002) and the Context Maintenance Retrieval Model (CMR) (Polyn et al., 2009), which were originally developed to explain retrieval clustering in list-learning paradigms.

According to these models, sequentially presented information is encoded against a slow drifting temporal context. Large contextual shifts—-such as task changes—-increases the drift rate, thereby separating the temporal contexts associated with different items. Consistent with these assumptions, paradigms based on temporal judgments (e.g., the EDD paradigm) show that items separated by an event boundary are perceived as being farther apart in subjective time than items within the same event. CMR further posits that, even when multiple contextual dimensions (e.g., semantic, perceptual) are present at encoding, temporal context will dominate retrieval organization. In contrast, the present gist-based findings suggest that while TCM and CMR adequately capture the principles underlying the within > across effect for temporal memory, they may not fully extend to content-based memory in naturalistic events. The reversed effect observed for gist details (within < across) indicates stronger associative links between semantic information from events separated by a boundary. This pattern suggests a mechanism by which narratives maintain continuity and coherence even as episodic details are segmented by event boundaries.

Importantly, not all naturalistic experiences require such contiguity. Zheng et al. (2022), for example, distinguish between *soft boundaries—*shifts between events within the same continuous narrative, as in the present study—and *hard boundaries*, such as transitions between entirely different video clips. Maintaining continuity across soft boundaries is crucial for narrative comprehension, whereas contiguity across hard boundaries is far less important. Future work comparing gist-based memory across situations that do or do not require narrative continuity could further test this account. Additional research is also needed to determine whether the integration of episodic information within events and the integration of gist information across events relies on shared or distinct neural mechanisms. Since we did not replicate the well-established within > across effect for overall accuracy, we are restricted in our scope for generalizing our conclusions. Although unlikely, it is possible that the results observed for the episodic and gist details may change if global memory accuracy reflects the effect. Therefore, it is imperative for future work to evaluate and replicate the current results, especially for gist information.

## 5 Conclusion

The current studies show that episodic and gist information are bound differently within vs. across events in naturalistic experiences. Importantly, this pattern was observed without any clear age-related differences in how event details are organized. Our results suggest that contiguity across events of an episode is maintained by associative links between semantic details. This mechanism for maintaining contiguity appears to operate similarly in young and older adults. Further, there is a possibility the widely reported within > across effect primarily reflects the organization of episodic features of an event.

## Data Availability

The original contributions presented in this study are included in the article/Supplementary material, further inquiries can be directed to the corresponding author.
