# Supplementary material for: Separating the details while maintaining the story: within event episodic integration and across event semantic contiguity in memory
Source: Front Psychol. 2026 Apr 28;17:1758774. doi: 10.3389/fpsyg.2026.1758774 (PMC13162987; doi:10.3389/fpsyg.2026.1758774)
Supplement: Supplementary file 1 [file Data_Sheet_1.pdf]

Supplementary Materials

Supplementary material 1: Normative segmentation study

The audio-visual stimulus used for encoding was a 35-minute video of the first episode of BBC's Sherlock titled 'Study in Pink'. To identify event boundaries in this clip, an independent sample of 50 participants (Mean age: 36.5; Mean education: 15.34; Female: 17) viewed and segmented the clip via the online platform Prolific (www.prolific.com). Participants watched the Sherlock episode and pressed a key whenever they perceived a meaningful event transition. Timestamps were converted to frame numbers and aggregated using a Gaussian kernel density estimate to create a continuous boundary-likelihood function across the video. Local maxima that met temporal separation criteria and exceeded a minimum response threshold were identified as candidate boundaries. This procedure produced 36 peaks one occurred too close to the end of the stimulus and was excluded, yielding a final set of 35 event boundaries used throughout the current study. The mean duration of these events was 52.11 seconds.

Two other studies using the same video as stimulus defined event boundaries either using scene cuts (Chen et al., 2016) or using a similar data-driven approach as ours (Silva et al., 2019) identified 33 and 38 event boundaries respectively. This suggests our event boundaries were comparable to other studies using the same movie.

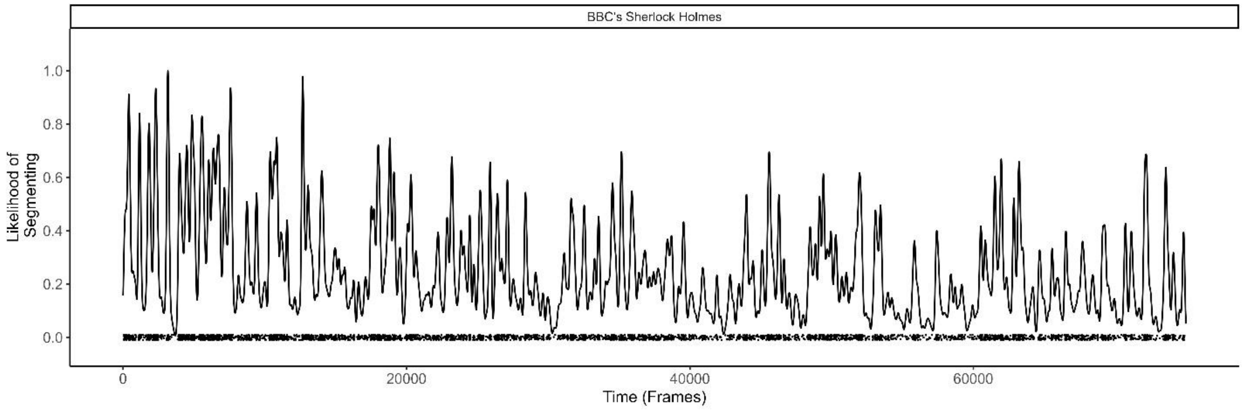

Figure S.1.1: Likelihood of an event boundary occurrence in any given frame of the BBC Sherlock video.

Supplementary Material 2: ***Experiment 1***

Psychometric tests:

In Experiment 1 participants were presented with computerized versions of the letter comparison task, pattern comparison task, and vocabulary task to assess comparability of both age groups.

Letter comparison: In the letter comparison task (Earles & Salthouse, 1995), participants were presented with pairs of non-word letter strings one at a time on a computer screen as a measure of processing speed. Participants' task was to indicate whether both strings were the same or different using the 'S' key and the 'D' key on the keyboard respectively. They were given 60 seconds to complete as many pairs as possible. The letter strings consisted of randomly selected consonants and ranged from 3 to 9 letters. Participants were instructed to respond as quickly and as accurately as they can.

Pattern comparison: In this task, participants compared pairs of patterns presented onscreen one at a time to judge whether the patterns were the same or different as a measure of processing speed. Response keys to indicate their response were the same as the letter completion task. This too was a timed task, and participants had to respond to as many pairs as possible within 60 seconds.

Shipley-Hartford vocabulary test: Participants completed a computerized version of the Shipley-Harford vocabulary test consisting of 37 target words as a measure of semantic knowledge. Each target word was presented along with 5 options, and their task was to select the option which was synonymous with the target word. Participants were asked to click on their selected option using a computer mouse to record their response. As commonly observed in studies with an aging population, older adults had significantly better mean vocabulary scores than YAs ($t = 3.16$, $df = 75.819$, $p < 0.01$).

Sherlock Familiarity: After completion of all tests, participants were asked to rate their familiarity with the episode of Sherlock they watched in the scanner (See Table S1.1 for Sherlock familiarity results).

Free recall task: Participants were seated at a different computer outside the scanner and were asked to type their response using a keyboard. They were instructed to recall the episode they watched, as if they were describing the episode to a friend who did not watch the video. They were instructed to recall the episode preserving the sequence of occurrences and in as much detail as possible. Participants were given 10 minutes to complete this task.

Table S2.1

Experiment 1: Participants' Prior Exposure to the Sherlock Episode Counts

	**Seen Several Times**	**Possibly Watched Once**	**Unsure if Ever Seen**	**Confident Never seen**	**Totals**
Older Adult	1	8	7	24	40
Younger Adult	3	5	8	23	37
Totals	4	13	15	47	77

Supplemental Material 3

Table S3.1

Binary Cued Recall Scoring

A rubric for binary scoring (remembered/forgotten) was developed by analyzing key actions/occurrences as described by (Chen et al., 2016). Participant responses were then scored appropriately if mentioning the key actions defined in the scoring rubric below.

Event Name	Major Event Action
1-Across	Watson opens his eyes/wakes up from his nightmare/ sits up breathing heavily/ lies back down and tries to calm himself/ cries
2-Within	Watson goes to his desk/sets down his mug/apple/sits down/opens a desk drawer and takes out his red laptop/ gun in drawer/ opens his laptop to his personal blog
3-Across	Man is lying on the floor/man is dead/The dead man's mistress, the secretary is upset, crying / camera/ Two men were walking in the rain. One of them went to bring an umbrella.
4-Within	Wife continues talking about dead husband/camera pans to woman or secretary or mistress of the dead man/ woman in purple blouse sheds a tear/ two young men walking in the rain sharing and umbrella/ one of them tries to hail a cab/ he goes back to get an umbrella
5-Within	All the reporters get texts saying 'wrong!'/Donovan asks the reporters to ignore the text they received/ Donovan asks if they have any more questions
6-Within	All the reporters get the third 'wrong!' text. /Lestrade receives 'You know where to find me SH'/ Donovan chastising Lestrade to make SH from stopping making the cops look like fools/ Lestrade's reply
7-Across	Watson and male friend sit on a park bench with coffee, catch up/ John can't afford London on an army pension. /Mike asks if Harry couldn't help/ Mike mentions getting a flat share (a roommate).
8-Within	Sherlock whips the dead body with a riding crop/ woman watches him from a window.
9-Within	Land lady opens the door for them/woman hugging sherlock, sherlock introduces Watson/ they go inside the house/ enter 221B
10-Across	A detective runs up the stairs to inform Sherlock of another murder/ Lestrade says that this time, they left a note and asked Sherlock to come. /He won't work with Anderson/Lestrade leaves.
11-Across	Conversation between landlady and Dr Watson/ Mrs. Hudson commenting on Sherlock's rushing everywhere/ Mrs. Hudson comparing Watson to her husband - the sitting down type/ Watson yelling in frustration & apologizing/ Watson reminding Mrs. Hudson about tea & biscuits/ Mrs. Hudson replying 'only once, not your housekeeper'/ Sherlock comes back into the room/ Sherlock asks John if he was a good army doctor and if he would like to join him
12-Within	Sherlock comes back into the room/ Sherlock asks John if he was a good army doctor and if he would like to join him
13-Within	Sherlock and Watson turn down a hallway to leave the house.
14-Within	Sherlock hails a taxi/ Taxi pulls up to the curb/ Both get into the cab and it drives away

15-Within	Sherlock explains to Watson what he observed about him in the lab (goes to flashback). Anything of the conversation from the taxi is scored correct.
16-Within	Sherlock and Watson arrive at the yellow tape, talk to the female cop/ Sherlock calls John a colleague/Sherlock implies in a conversation with Anderson that him and Sally slept together./ Sherlock and John walk into the building/ They enter a room and meet Lestrade/ Lestrade asks who Watson is, Sherlock introduces as a colleague/ Lestrade & Watson put on PPE, Sherlock refuses.
17-Within	Lestrade says he can only give some time to Sherlock to check the crime scene/ The camera pans to the dead body/ they continue upstairs where dead body is/ they reach the dead body
18-Across	Sherlock leaves, the detective and Watson are left alone/ Watson turns around/ Lestrade moves towards room/ Watson starts limping/climbing down the stairs, bumps into someone, gets frustrated/ Watson exits the building.
19-Across	Watson walks away from ringing phone and reaches the main road.

Table S3.2

Cued Recall Task: Results of Generalized Linear Mixed Effects Model for Remembered vs Forgotten from Experiment 1

Accuracy ~ Age group + Cue type + (1|Cue clip) + (1|Participant)				
	**Estimate**	**Standard error**	**z. value**	**Pr(>|z|)**
Intercept	-0.721	0.423	-1.704	0.088
Cue Type (within)	0.009	0.480	0.018	0.985
Age (Young)	1.761	0.272	6.468	< .001 ***

Table S3.3

Estimated Marginal Means for Cued Recall Accuracy (Remembered vs Forgotten) by Age Group

Predictor	Estimated marginal means	SE	Confidence interval (lower)	Confidence interval (upper)
Old	0.328	0.066	0.213	0.468
Young	0.740	0.058	0.612	0.836
Across	0.540	0.099	0.349	0.720
Within	0.542	0.077	0.390	0.686

Table S3.4

Cued Recall Task: Result of Generalized Linear Mixed Effects Model for Remembered vs Forgotten (best fit model)

Accuracy ~ Age group + (1|Cue clip) + (1|Participant)				
	**Estimate**	**Standard error**	**z. value**	**Pr(>|z|)**
Intercept	-0.71	0.29	-2.440	0.0147*
Age (YA)	1.76	0.27	6.468	9.91e-11***

Table S3.5

Estimated Marginal Means for the Best Fit Model for the Cued Recall Task (reported in Table S5)

Age	Predicted probability	SE	df	asymp.LCL	asymp.UCL
OA	0.328	0.065	Inf	0.216	0.465
YA	0.740	0.056	Inf	0.616	0.835

Supplemental Material 4

Cued Recall Episodic and Semantic Scoring

In the original scoring scheme (Levine et al., 2002), internal (or episodic) details include the following categories: events (actions or occurrences), time, place (location of the event), perceptual (sensory details) and emotion/thought. The external (gist) details include the following categories: semantic (general knowledge, facts, background information), repetition, other (metacognitive statements, editorializing).

Here, we outline the modifications to the categories included in the original scoring scheme to adapt the scoring for recall of verifiable episodic memories. First, the 'external event' category from the original scoring rubric was omitted, as participants were not expected to recall other episodes of BBC's *Sherlock* or other unrelated details from their life when instructed to only respond with "what happened immediately after the clip?". Second, the 'Semantic' category scoring included paraphrased or summarized descriptions of actions or occurrences within the target event, as well as factual statements about the video. This was defined as any action or occurrence which is mentioned in the correct response rubric but is paraphrased. Third, repetition category was specifically defined as any repetition of details from the cue clip or an already mentioned detail. Fourth, an additional category titled 'Guess' responses was included in the tally of external details. This category identified details in responses based on schema information extracted through the cue clip even without watching the whole video. The rubric for the schema-based responses was developed using data from a separate online experiment with a different cohort of participants (9 participants; Mean age: 38.55 yrs; Gender: 1 male, 8 females; Ethnicity: 1 black, 8 whites; Country of origin: 1 UK, 8 US). The experiment was developed using PsychoPy v2024.2.0 and hosted online via Pavlovia. These participants completed the same cued recall task as those in the main experiment but did not watch the *Sherlock* video beforehand. As a result, their responses reflected only their pre-existing schemas or those developed by viewing the cue clips rather than memory for the video. For each cue-clip, occurrences/actions that were mentioned by more than 6 participants were formulated as the 'Guess' response for that cue-clip. While scoring participants' responses in the main experiment for the 'Guess' category each clause was compared to these schema-based responses; any clause that matched was coded as a 'Guess' detail.

Each response for a cue clip was first parsed into clauses. Then each clause was compared to the clauses in three rubrics. If the clause did not match the correct response rubric, it was scored as 'Incorrect'. If it matched the cue rubric, it was scored as 'Repetition'. If it matched the schema-based rubric, it was scored as 'Guess'. If a clause was scored in either of these categories, their details were excluded from being scored for the remaining categories. The model updated the tallies for each row in the csv file provided. Because row-by-row scoring in the chat produced more consistent results than batch processing, the row-wise scoring approach with manual monitoring of ChatGPT's responses was used (See Figure S2 for example prompt scoring).

Participant's response / Prompt:

"Gun shots ring, Watson opens his eyes. He wakes up in a dark room early in the morning from a nightmare scared. He was shot during the fighting. He spent time recovering from his dream. All around a scary experience."

Description of Cue clip: Scene of a battlefield or war. Soldiers voices echoing. Sound of guns shooting.

Sample correct response: John/Watson opens his eyes. Wakes up from his nightmare and sits up on his bed. He is breathing heavily. Then lies back down and tries to calm himself. he starts weeping.

Sample schema predicted response: The war fighting will continue. Any speculations about the war

Clause Break Down:

Clause 1: **"Gun shots ring,"**
Score: "Gun shots ring"- **Repetition** (Included in Cue)

Clause 2: "*Watson opens his eyes.*"
Score: "Watson opens his eyes"- *Event*, *Perceptual*

Clause 3: "*He wakes up in a dark room early in the morning from a nightmare scared.*"
Score: "He wakes up in a dark room early in the morning from a nightmare scared"- 1x *Event*
"Dark Room"- 1x *Perceptual*, 1x *Place*
"Early in the morning"- 1x *Time*
"Scared"- 1x *Emotion/Thought*

Clause 4: **"He was shot during the fighting."**
Score: "He was shot during the fighting."-1x **Guess** (Included in the Schema Prediction)

Clause 5: **"He spent time recovering from his dream."**
Score: "He spent time recovering from his dream"-1x **Semantic**

Clause 6: **"All around a scary experience."**
Score: "All around a scary experience."-1x **Other** (Editorializing)

Final Prompt Score:

- *Event x2*
- *Perceptual x2*
- *Time x1*
- *Emotion/Thought x1*
- *Place x1*
- **Repetition x1**
- **Guess x1**
- **Semantic x1**
- **Other x1**

Figure S4.1. The figure illustrates the clause break down process and example scoring categories. Episodic details are shown in green italics, and Gist details are shown in yellow **boldface.**

Table S4.1

Experiment 1: Generalized Linear Mixed Effects Model Results for Episodic Detail Recall

Episodic detail counts ~ Cue type * Age + (1|Cue clip) + (1 + Cue type | Participant)				
	**Estimate**	**SE**	**z. value**	**Pr(>|z|)**
Intercept	0.159	0.149	1.067	0.286
Cue type (within)	0.408	0.122	3.350	< .001 ***
Age group (Young)	0.797	0.141	5.645	< .001 ***
Cue type × Age group (Young)	-0.278	0.134	-2.072	.038 *

Table S4.2

Experiment 1: Estimated Marginal Means for Episodic Detail Recall

Cue Type	Age Group	Response	SE	Confidence interval (Lower)	Confidence interval (Upper)
Across	Old	1.170	0.174	0.876	1.569
Within	Old	1.760	0.237	1.354	2.292
Across	Young	2.600	0.327	2.032	3.328
Within	Young	2.960	0.347	2.351	3.328

Table S4.3

Experiment 1: Generalized Linear Mixed Effects Model Result for the Gist Detail Recall

Gist detail counts ~ Cue type + Age + (1 + Cue type | Participants) + (1| Cue clip)				
	**Estimate**	**SE**	**z. value**	**Pr(>|z|)**
Intercept	0.442	0.117	3.769	< .001 ***
Cue type (within)	-0.483	0.075	-6.421	< .001 ***
Age group (Young)	0.127	0.093	1.366	0.172

Table S4.4

Experiment 1: Estimated Marginal Means for Gist Detail Recall

Predictor	Response	SE	Confidence interval (Lower)	Confidence interval (Upper)
Across	1.658	0.171	1.354	2.030
Within	1.022	0.106	0.834	1.253
Old	1.222	0.136	0.983	1.519
Young	1.388	0.142	1.135	1.697

Supplemental Material 5: Experiment 2

Psychometric Tasks

Psychometric Battery: Participants also completed cognitive assessments using the NIH Toolbox on an iPad with the researcher. Before starting the session, the researcher registered each participant on the iPad and entered their demographic information, including date of birth, sex, race, and education level. Using the NIH Toolbox, we assessed various cognitive domains: learning and memory with the Rey Auditory Verbal Learning Test (RAVLT), processing speed with the Pattern Comparison Processing Speed Test, executive function and attention with the Flanker Inhibitory Control and Attention Test, and language and vocabulary knowledge with the Picture Vocabulary Test. Norms for the NIH Toolbox were created based on a sample of 4,859 participants, aged 3 to 85 years, reflecting the diversity of the U.S. population in terms of age, gender, race/ethnicity, and education. Standardized scores, such as Fully Corrected T-Scores, are calculated using this normative data and adjusted for demographic factors, enabling meaningful comparisons within similar groups (National Institutes of Health (NIH) Toolbox, 2021) (See Table S5.1 for age group comparison).

Rey Auditory Verbal Learning Test (RAVLT): Episodic long-term memory was assessed using the RAVLT. Participants were asked to learn a list of 15 words presented verbally. Care was taken to ensure the volume was turned up loud enough for participants to hear. Participants first listened to the word list and then recalled as many words as possible. The correct responses were listed on the iPad screen that the researcher held, ensuring that the participants could not see. As the participant recalled the words on the list, the researcher touched the corresponding word on the screen to log the response. Only correct responses were recorded. If the participant recalled an incorrect word or repeated a response, this was not recorded. This encode-recall process was repeated three times to produce an immediate memory score. After a delay of approximately 10 minutes, participants were tested again, this time without hearing the list again. The total time for this task was 4 minutes. This task yields an immediate and a delayed test score.

Pattern Comparison Processing Speed Test: Participants completed the Pattern Comparison Processing Speed Test on their own using the iPad. This task assessed processing speed by asking participants to quickly determine whether two presented stimuli were the same or different. Participants were shown pairs of images and instructed to decide whether the images were the same or different while moving through the task as quickly as possible without making mistakes. Participants completed as many trials as possible within 4 minutes.

Flanker Inhibitory Control and Attention Test: The Flanker Inhibitory Control and Attention Test assessed participants' ability to focus on a central target while ignoring distractions. Participants were shown five fish with arrows on them. Their task was to determine the direction of the arrow on the center fish while ignoring the “flanker” fish on either side. The arrows on the flanker fish sometimes pointed in the same direction as the center fish (congruent trials) and sometimes pointed in the opposite direction (incongruent trials). This task measured participants'

ability to maintain focus on a specific target while filtering out irrelevant distractions. The total time for this task was 4 minutes.

Picture Vocabulary Test: The Picture Vocabulary Test assessed participants' vocabulary knowledge. For each trial, participants listened to an audio recording of a word and were shown four pictures. Their task was to select the picture that most closely matched the meaning of the word. The total time for this task was 3 minutes.

Sherlock Familiarity: Before participating in Experiment 2, individuals completed a survey assessing their familiarity with several popular television shows, including *BBC's Sherlock*. Participants who indicated prior exposure to *Sherlock* were excluded from the study.

Free recall task: Participants were then escorted to a different laboratory computer and were asked to type their response using a keyboard. They were instructed to recall the episode they watched, as if they were describing the episode to a friend who did not watch the video. They were instructed to recall the episode preserving the sequence of occurrences and in as much detail as possible. Participants were given 15 minutes to complete each response.

Table S5.1

Comparison of Young and Older Adults Across NIH Toolbox Tests for Experiment 2

	Young adults		Older adults		*t*	*f*	*p*
	M	*SD*	*M*	*SD*			
NIH toolbox Pattern Comparison Processing Speed	48.7	10.87	49.9	10.07	0.51	0.275	.60
Picture Vocabulary	37.3	10.14	50.5	7.08	6.52	44.31	.001***
Flanker Inhibitory Control & Attention	51.4	10.09	50.0	6.90	-0.66	.45	.50
RAVLT (Immediate)	51.3	9.35	54.8	11.88	1.45	2.403	.13
RAVLT (delayed)	56.6	8.77	60.8	8.77	2.28	5.712	.02*

Event Memory Tasks

(See Table S5.2 for age group comparison).

Recognition Memory Task: Recognition memory was measured using a 20-trial, two-alternative forced-choice (2AFC) paradigm. On each trial, participants viewed two static images presented side-by-side. The images were closely matched in visual content (e.g., the same

character, setting, and objects). One image was drawn from the 35-minute portion of *Sherlock* that participants had watched, and the other was taken either from a later, unseen portion of the same episode or from a different episode entirely. Participants indicated which image they recognized from the viewed segment by pressing the corresponding left or right arrow key. Trials were randomized and counterbalanced across participants. Accuracy was scored on a binary scale (1 = correct, 0 = incorrect).

Temporal Order Memory Task: Temporal order memory was assessed using a 20-trial 2AFC task modeled after the recognition task but with a different decision requirement. In each trial, two images from the viewed portion of the episode were presented, and participants judged which image appeared earlier in the narrative. Responses were made using the arrow keys, and all trials were randomized and counterbalanced across participants. Accuracy was scored as correct or incorrect for each trial.

Table S5.2

Comparison of Young and Older Adults Across Event Memory Tests for Experiment 2

	**Young adults**		**Old adults**		*t*	*f*	*p*
	M	SD	M	SD			
Recognition	0.86	0.118	.86	0.09	-0.34	0.115	.73
Recognition Reaction Time	5.56	2.25	6.14	3.07	1.27	1.587	.22
Temporal Order	0.69	0.166	.66	0.154	-0.99	0.912	.34
Temporal Order Reaction Time	5.24	1.84	7.42	2.74	4.71	23.194	.001***

Table S5.3

Experiment 2: Generalized Linear Mixed-Effect Model Results for Cued Recall Accuracy

**Accuracy ~ Age group + Cue type + (1|Cue clip) + (Cue type |Participant)**				
	**Estimate**	**Standard error**	**z. value**	**Pr(>|z|)**
Intercept	−0.046	0.211	-0.218	0.83
Cue Type (Across)	−0.117	0.182	−0.642	0.52
Age (Old)	−0.344	0.123	−2.794	.005**

Table S5.4

Experiment 2: Estimated Marginal Means for Cued Recall Accuracy

Predictor	Estimated marginal means	SE	Confidence interval (lower)	Confidence interval (upper)
Old	0.404	0.059	0.295	0.522
Young	0.574	0.060	0.455	0.685
Across	0.459	0.077	0.317	0.609
Within	0.518	0.062	0.398	0.635

Table S5.6

Experiment 2: Generalized Linear Mixed Effects Model Results for Episodic Details

Episodic detail count ~ Cue type + (1 + Cue type | Participant)				
	**Estimate**	**SE**	**z. value**	**Pr(>|z|)**
Intercept	0.908	0.120	7.562	< .001***
Cue type (within)	-0.082	0.054	1.513	0.13

TableS5.7

Experiment 2: Estimated Marginal Means of Episodic Details Across the two Cue Types

Cue type	Estimate	SE	df	Confidence Interval (Lower)	Confidence Interval (Upper)
Across	2.479	0.30	Inf	1.10	3.13
Within	2.691	0.30	Inf	2.17	3.34

Table S5.8

Experiment 2: Generalized Linear Mixed Effects Model Results for Gist Details Recalled

Gist detail count ~ Cue type + Age group + (1 + Cue type | Participants) + (1| Cue clip)				
	**Estimate**	**SE**	**z. value**	**Pr(>|z|)**
Intercept	0.226	0.141	1.589	0.112
Cue type (within)	-0.332	0.078	-4.177	< .001 ***
Age group (Young)	-0.094	0.098	-0.955	0.339

Table S5.9

Experiment 2: Estimated Marginal Means for Gist Details Recalled

Predictor	Response	SE	Confidence interval (Lower)	Confidence interval (Upper)
Across	1.193	0.155	0.925	1.540
Within	0.862	0.105	0.679	1.090
Old	1.063	0.140	0.821	1.370
Young	0.968	0.123	0.754	1.240

Supplemental Material 6

Total count of Episodic and Gist-Based Details Recall Analysis

One aim of the study was also to compare whether the within > across effect is uniformly observed for both the episodic and gist details. The results reported in the main manuscript include a qualitative comparison of this difference since episodic and gist details were never statistically compared. Also the statistical comparisons reported in the main manuscript always included the influence of age. To statistically compare the difference in within > across effect for the two types of event details, without age influencing the effect a generalized mixed model analysis with a negative binomial distribution was conducted for total count of episodic and gist details for both Experiment 1 and Experiment 2.

For both experiments, the full interaction model specified before model comparisons included Detail type (Episodic / gist) and Cue type (within /across) as fixed effects. A random slope intercept for Participants by Detail type to control for variation in estimates due to age or individual participants, a random intercept for cue clips proved to be the maximal random effects structure justified by the data and were specified as random effects in the model. This full interaction model was compared to reduced models to find the model with the best maximal effects structure for the observed data. Further results are discussed below for each experiment separately –

Experiment 1: Total count of episodic and gist-based details recalled

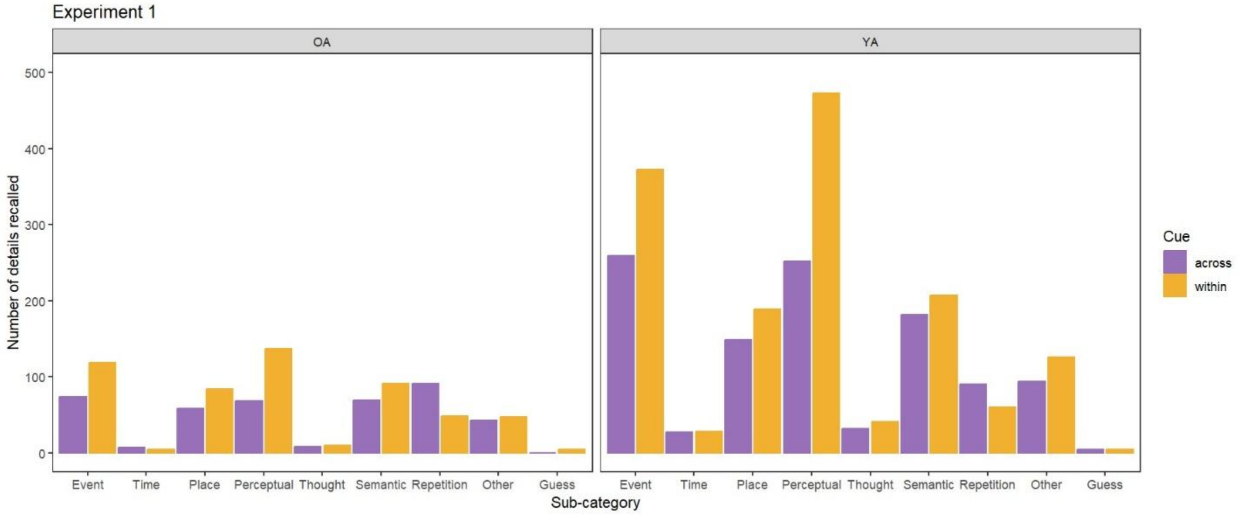

Figure S6.1 Raw counts of number of details were recalled in each sub-category of Episodic and Gist categories in Experiment 1

Model comparison suggested that the full interaction model had a significantly better fit than the reduced models and had the lowest AIC. Results showed a significant interaction between Detail type and Cue type, $\beta = 0.42$, SE = 0.08, z value = 5.03, $p < .001$; a significant main effect of Detail type, $\beta = 0.31$, SE = 0.09, z value = 3.65, $p < .001$, and a significant main effect of Cue type, $\beta = -0.30$, SE = 0.07, z = -4.25, $p < 0.01$.

Post hoc analysis indicated that the number of episodic details reported from within an event (emm = 2.26, SE = 0.23, df = Inf, CI = 1.84 to 2.76) were not significantly greater than those reported across an event (emm = 2.00, SE = 0.22, df = Inf, CI = 1.61 to 2.47), ratio = 0.885, SE = 0.05, df = Inf, z.ratio = -2.13, p = 0.14. But a significantly greater number of gist-based details were reported from across an event (emm = 1.45, SE = 0.14, df = Inf, CI = 1.19 to 1.76) as compared to from within an event (emm = 1.07, SE = 0.10, df = Inf, CI = 0.89 to 1.29), ratio = 1.35, SE = 0.09, df = Inf, z.ratio = 4.25, $p < 0.001$. Consistent with prior studies we observed a within > across pattern; albeit non-significant; for episodic details but a reversed (within < across) effect for External details. These results are consistent with the results reported in the main manuscript including the predictor of age. Thus, the lack of a significant within > across effect cannot be simply attributed to lower power to detect the effect due to the presence of another predictor. Further, the total number of episodic details recalled were significantly greater than the total number of gist details in both the within (Episodic: emm = 2.26, SE = 0.23, df = Inf, CI = 1.84 to 2.76; Gist emm = 1.07, SE = 0.10, df = Inf, CI = 0.89 to 1.29; ratio = 0.47, SE = 0.03, df = Inf, z.ratio = -8.97, $p < 0.0001$) and across (Episodic: emm = 2.00, SE = 0.22, df = Inf, CI = 1.61 to 2.47; Gist emm = 1.45, SE = 0.14, df = Inf, CI = 1.19 to 1.76; ratio = 0.728, SE = 0.06, df = Inf, z.ratio = -3.65, $p < 0.01$) conditions.

Experiment 2: Total number of episodic and gist-based details recalled

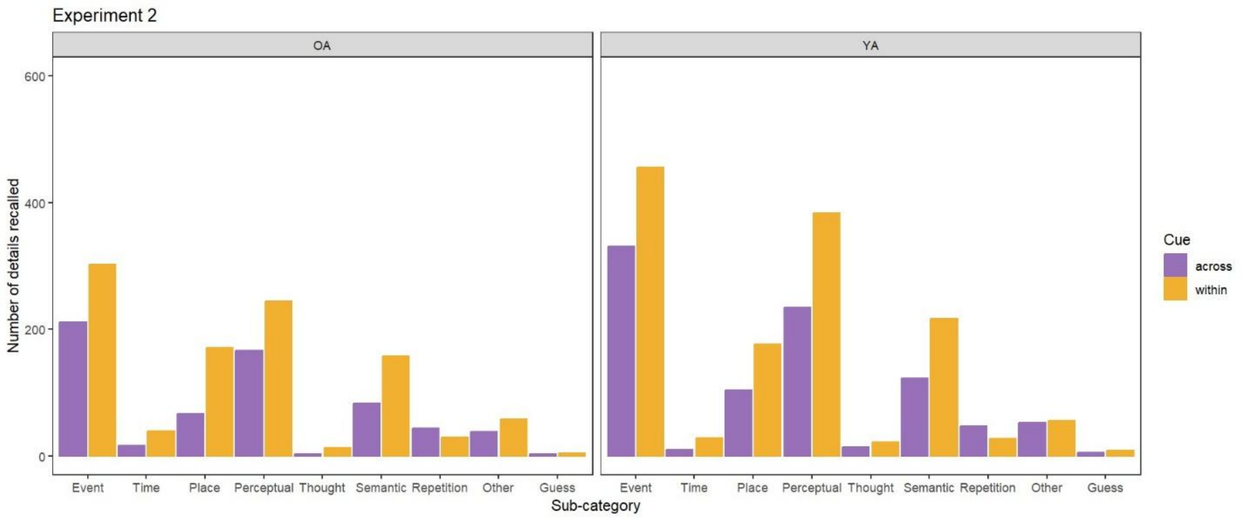

Figure 6.2 Raw counts of number of details were recalled in each sub-category of Episodic and Gist categories in Experiment 2

Results of model comparison showed the full interaction model had the lowest AIC than the other models. Results showed the interaction between Detail type and Cue type to be non-significant, $\beta = 0.13$, SE = 0.08, z value = 1.59, $p = 0.11$. Both main effects of Cue type ($\beta = -0.15$, SE = 0.07, z = -2.22, $p < 0.05$) and Detail type ($\beta = 1.06$, SE = 0.06, z value = 17.22, $p < 0.001$) were significant.

Post hoc analysis showed an advantage for remembering episodic details in both the within-event, (episodic: emm = 2.70, SE = 0.20, df = Inf, CI = 2.41 to 3.22; gist-based: emm = 0.85, SE = 0.06, df = Inf, CI = 0.72 to 0.99), ratio = 0.30, SE = 0.01, df = Inf, z.ratio = -23.55, $p < 0.001$ as well as the across-event trials, (episodic: emm = 2.60, SE = 0.47, df = Inf, CI = 1.82 to 3.74; gist-based: emm = 0.90, SE = 0.16, df = Inf, CI = 0.62 to 1.30), ratio = 0.35, SE = 0.02, df = Inf, z.ratio = -17.22, $p < 0.001$, indicating an overall better memory for remembering episodic details as compared to gist based details. Further for the effect of Cue type, neither the episodic (Within: emm = 2.79, SE = 0.22, df= Inf, CI = 2.38 to 3.27; Across: emm = 2.88, SE = 0.24, df = Inf, CI = 2.43 to 3.41) nor the gist-based details (Within: emm = 0.85, SE = 0.07, df = Inf, CI = 0.71 to 1.01; Across: emm = 0.99, SE = 0.09, df = Inf, CI = 0.82 to 1.20) showed a within > across effect (Internal: ratio = 1.03, SE = 0.04, df = Inf, z.ratio = 0.66, p =0.90; External: ratio = 1.172, SE= 0.08, df = Inf, z.ratio = 2.22, p = 0.11).

In both experiments we do not observe a within > across for episodic details. But instead, we found a reversed advantage for remembering gist-based details across events only in Experiment 1 but not in Experiment 2. However, results reported in the main manuscript, including the influence of age indicated a reversed, i.e., within < across for gist-based details for both Experiments 1 & 2. Because gist-based memories are not susceptible to age-related decline (cite) and older adults possibly also have better schema memories (cite), there is a possibility the reversed advantage for across memories is driven by age-related differences in event representations.

Supplementary Material 7

The reversed within vs across effect observed for gist details across experiments 1 and 2 could be due to greater causal connection between cue and target that belonged to two different events – across event trials – as compared to within event trials. To ensure that there were equal causal connections between cue and target irrespective of whether it was a within or across trials we used the criteria specified by van den Broek (1990) to categorize the target responses as causal or non-causal. Descriptive statistics for accuracy, causal connectedness and Cue type are specified in Table 7.1 (Experiment 1) and Table 7.2 (Experiment 2)

Table 7.1

Experiment 1: Mean number of details recalled for the two types of trials as a function of causal connectedness between Cue and Target

Age	Causal Connectivity	Trial type	Gist-based details	Episodic details
OA	no	across	1.69 (0.90)	2.13 (1.78)
YA	no	across	2.03 (1.20)	4.07 (2.88)
OA	yes	across	1.59 (0.73)	1.53 (1.39)
YA	yes	across	1.72 (0.60)	3.30 (1.66)
OA	no	within	0.93 (0.60)	2.18 (1.29)
YA	no	within	1.04 (0.49)	3.18 (1.64)
OA	yes	within	1.25 (0.86)	1.66 (1.29)
YA	yes	within	1.42 (0.98)	3.57 (1.84)

Table 7.2

Experiment 2: Mean number of details recalled for the two types of trials as a function of causal connectedness between Cue and Target

Age	Causal Connectivity	Trial type	Gist-based details	Episodic details
OA	no	across	1.12 (0.74)	3.82 (1.91)
YA	no	across	1.08 (0.68)	4.52 (2.37)
OA	yes	across	1.41 (0.88)	2.91 (1.87)
YA	yes	across	1.35 (0.84)	3.07 (2.43)
OA	no	within	0.97 (0.84)	3.47 (1.16)
YA	no	within	0.77 (0.54)	3.69 (1.55)
OA	yes	within	1.11 (0.79)	2.88 (1.49)
YA	yes	within	0.99 (0.58)	2.49 (1.14)
