# Supplementary material for: Separating the details while maintaining the story: within event episodic integration and across event semantic contiguity in memory
Source: Front Psychol. 2026 Apr 28;17:1758774. doi: 10.3389/fpsyg.2026.1758774 (PMC13162987; doi:10.3389/fpsyg.2026.1758774)
Supplement: Supplementary file 2 [file Supplementary_file_1.pdf]

Supplementary Material: Prompts for image generation

Image 1: Generate a zoomed out scene showing the pavement outside 221B with 4-5 people walking and one of the Figures from Image 2 I provided walking towards 221B.

Image 2: Create one image where one cartoon figure is shaking hands with another cartoon figure in front of a closed door with the number 221 B written on the door. Recreate this as a minimal illustration that one would see in a newspaper comic strip

Image 3: create an image that shows one of the characters entering through the door to 221B while the other character is about to follow him inside. The view can be profile view.

Image 4: now just a closed door of 221B with the street outside.

Image 5: create an image showing the person with the bag climbing a staircase inside a house. The viewpoint is from the top of the staircase. Lose the bag. Show the person using a cane while climbing and a subtle expression of struggle on his face.

Image 6: create a new image with the man with the scarf standing at the top of the stairs at the landing with the subtle expression of him waiting for the man with the cane to climb up. The viewpoint is from the bottom of the stairs.

Image 7: Create new image showing both of them have reached the landing and standing in front of an open door, about to enter that room

Image 8: create an image that shows one of the characters entering through the open door into the room inside while the other character waits to enter through the door."
